# S100A8/9-NLRP3-mediated chronic unresolved inflammation drives cardiac pathologies following invasive pneumococcal disease

**DOI:** 10.1038/s12276-025-01552-8

**Published:** 2025-10-10

**Authors:** Sultan Tousif, Daniel Minassian, Chao He, Baldeep Singh, Prachi Umbarkar, Arvind Singh Bhati, Mohammed Mohasin, Nathan Erdmann, Min Xie, Palaniappan Sethu, Carlos J. Orihuela, Hind Lal

**Affiliations:** 1 Department of Cellular Biology and Anatomy, LSUHS, LSU Health Shreveport, Shreveport, LA USA; 2 Department of Microbiology, Heersink School of Medicine, The University of Alabama at Birmingham, Birmingham, AL USA; 3 Division of Pulmonary, Allergy and Critical Care Medicine, The University of Alabama at Birmingham, Birmingham, AL USA; 4 Division of Cardiovascular Disease, The University of Alabama at Birmingham, Birmingham, AL USA; 5 Division of Infectious Diseases, Department of Medicine, The University of Alabama at Birmingham, Birmingham, AL USA

**Keywords:** Chronic inflammation, Heart failure

## Abstract

*Streptococcus pneumoniae* (*Spn*) is the leading cause of community-acquired pneumonia (CAP). A quarter of hospitalized patients with CAP experience a major adverse cardiac event (MACE), raising their mortality by four to five times compared with pneumonia alone. Patients with CAP continue to face a significantly greater risk of MACE and cardiovascular-associated death during convalescence. However, the reasons responsible for this remain unclear. To elucidate the molecular mechanism(s) of *Spn*-induced MACE in convalescence, a mouse model of *Spn* infection and antibiotic rescue was employed. A marked decline in ejection fraction persisting at least 3 weeks after bacterial eradication with antibiotics was observed. Evidence of enduring cardiac injury was observed at the molecular, biochemical and histology levels. Blood analysis from patients with invasive pneumococcal disease confirmed unresolved inflammation in these individuals. Here we mechanistically identified that S100A8/A9-TLR4-NLRP3-mediated unresolved inflammation drives cardiac pathologies in *Spn* convalescent mice. This inflammation was central to the cardiac pathology because interventions with broad-spectrum immunosuppressive hydrocortisone or specific inhibitors of S100A9 (paquinimod) essentially rescued the *Spn*-induced cardiac pathologies. These results provide critical preclinical data and rationale for a clinical investigation into immunosuppressive interventions for managing *Spn*-mediated cardiac pathologies in convalescence.

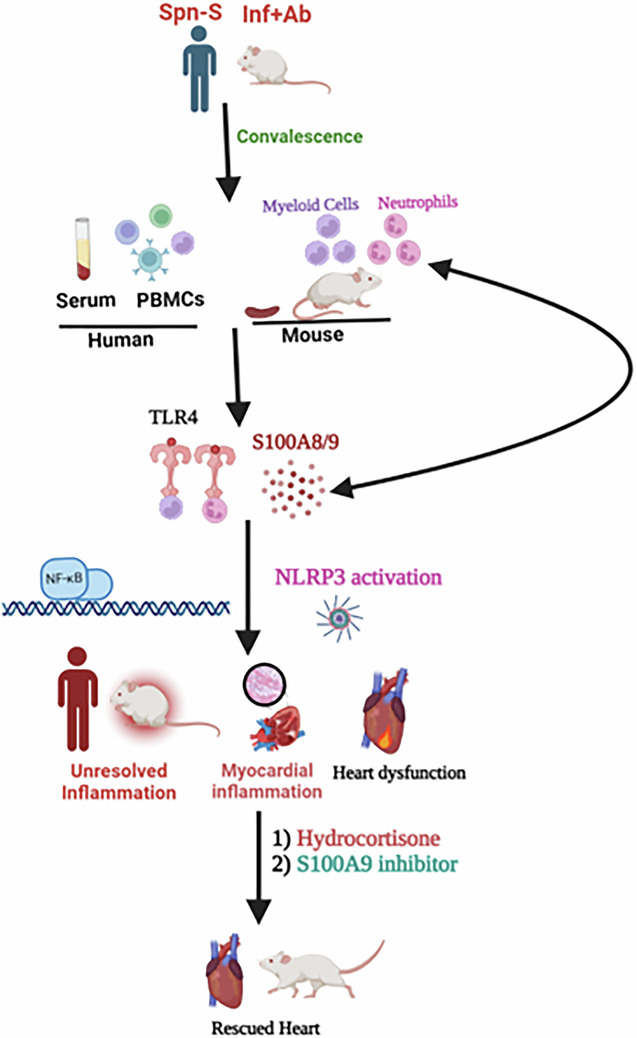

## Persistent Cardiac Injury After Pneumonia Revealed in Study

Hospitalization for community-acquired pneumonia (CAP) can lead to serious heart problems, even after recovery. Researchers explored why this happens. They studied both humans and mice to understand the link between pneumonia and heart problems. The study involved 10 healthy adults and 7 pneumonia patients. Researchers collected blood samples to analyze immune responses. They also used mice to study heart changes after pneumonia. The focus was on inflammation and its role in heart damage. Findings showed that inflammation persists even after the infection clears, leading to heart issues. The study identified a specific inflammatory pathway (S100A8/A9-TLR4-NLRP3) as a key player in this process. The researchers concluded that targeting this inflammation could help prevent heart problems after pneumonia. Future treatments might focus on reducing inflammation to protect the heart in pneumonia survivors.

## Introduction

Hospitalization for community-acquired pneumonia (CAP) is a notable risk factor for major adverse cardiac events (MACE), including heart failure (HF)^1–4^. While as many as one in four individuals hospitalized for CAP experience MACE, a heightened risk for MACE persists well beyond discharge^1^. Clinical investigations on post-CAP MACE have demonstrated that adults with CAP were two- to sevenfold more likely to develop new-onset HF within the first 3 months post hospitalization and about twice as likely to develop HF 5–10 years post hospitalization, even after adjusting for the presence of cardiovascular risk factors^1,5^. Moreover, serum levels of cardiac troponin-T, a marker of cardiac damage, at the time of hospital admission were found to be strong predictors of mortality in the years that followed. Thus, post-CAP MACE is a major driver of morbidity and mortality^1,2^.

While the onset of MACE during acute pneumonia is probably influenced by the physiologic stress imposed by the infection, microbial products in the circulation, a prothrombotic inflammatory state and the medications used to treat the infection, the underlying causes of post-CAP MACE are probably connected but also distinct^6–8^. Along such lines, heart sections from nonhuman primates intratracheally challenged with *Streptococcus pneumoniae* (*Spn*), the leading cause of CAP, showed considerable cardiomyopathy during acute infection. In turn, heart sections from baboons rescued from pneumonia with intravenous ampicillin demonstrated prolific immune cell infiltration into the heart and de novo collagen deposition/cardiac scar formation 1 week after the onset of antibiotic treatment. These animals also displayed diffuse, nonspecific repolarization abnormalities in ECG (electrocardiogram)^9,10^. Similar results have been obtained using experimentally challenged mice. In this instance, *Spn*-infected, ampicillin-rescued mice demonstrated disease-severity-dependent levels of collagen deposition and compromised cardiac function at 3 months post infection^9^.

Cardiac remodeling following sterile injury involves multiple cell types with substantial interplay between cardiomyocytes, fibroblasts and immune cells^11^. Across numerous cardiac disease conditions such as ischemic cardiomyopathy, myocarditis and hypertensive cardiomyopathy, unresolved inflammation has increasingly been implicated in contributing to pathology^11–13^. Inflammation and pathological remodeling are also features of chronic infections of the heart, such as Q-fever and Chaga’s disease^14,15^. During infection, the release of damage-associated molecular patterns (DAMPS), such as S100A8/9, together with the presence of pathogen-associated molecular patterns, leads to activation of Toll-like receptors (TLR), infiltration of innate immune cells and recruitment of adaptive immune cells which promote further infiltration of myeloid cells^16,17^. Unresolved infections also result in cardiac remodeling and cardiac dysfunction^7,18^. Critically, the immunological events that take place in the heart following an infectious insult, such as those that occur following successful antibiotic treatment for severe CAP, remain uncharacterized. This is a major knowledge gap given the frequency of hospitalizations for pneumonia, its association with MACE in convalescence, and the estimated US mortality rate of 30.6% 1 year following diagnosis^19^.

Clinical guidelines have recently been updated to support the use of immunomodulation by steroids in patients hospitalized with severe CAP. This comes following evidence that hydrocortisone treatment in severe CAP improved long-term mortality^20,21^, yet it is unclear whether immunomodulation during treatment for severe CAP-associated infections prevents adverse cardiac remodeling or MACE in convalescence, as well as whether inflammation plays a causal role in post-CAP MACE. Although efforts to use immunotherapy to treat cardiac disease have thus far been mixed, successes in particular groups of patients hint at the promise of such approaches and are suggestive that a similar approach may be taken with regard to preventing post-CAP MACE^12,22–24^. However, this requires that the inflammatory components bearing responsibility for post-CAP MACE be identified so that they can be specifically targeted. Here, we leveraged our well-characterized mouse model of invasive pneumococcal disease (IPD), a common feature of severe *Spn* infection, and antimicrobial rescue for comprehensive immune analysis to determine the specific mechanism of persistent unresolved inflammation in *Spn*-mediated cardiac pathologies in convalescence. Moreover, we tested the consequence of broadly and specifically blocking inflammatory S100A8/9-TLR4-NLRP3 inflammatory cascade. Our results advance the understanding of events that occur in the heart in convalescence for CAP and offer a promise of potential therapeutic intervention to prevent post-CAP MACE.

## Materials and methods

### Research participants and sampling

A total of ten healthy adult volunteers and seven adult subjects patients with pneumonia and IPD (male and female) were recruited in this study. A total of eight serum samples from healthy volunteers and seven from patients with pneumonia were used for cytokine and chemokine assays. However, some samples did not yield the results and were excluded from the graph. We matched patients with pneumonia versus controls for age and sex. We did not recruit any patients with underlying cardiovascular diseases or patients with underlying cardiovascular diseases are excluded. Patients had repeated blood cultures to ensure clearance of bacteremia (that is, negative blood culture). The study was approved by UAB (IRB-30000924), and those who participated in these studies were enrolled in the department of medicines at Birmingham, USA. The study was approved by the UAB Institutional Review Board (IRB-30000924 and 300010895). The clinical characteristics of the subjects are provided in Supplementary Table 1.

### Isolation of PBMCs and serum

Peripheral blood mononuclear cells (PBMCs) were isolated by density gradient centrifugation with Ficoll-Paque PLUS density gradient media (GE Healthcare) from about 9 ml of peripheral blood as previously described^25^. Freshly isolated PBMCs were utilized for the following flow cytometry analysis. The plasma serum was collected into a new 1.5 ml tube separated and stored at −80 °C for future experiments.

### Human antibodies/kits

From BD Pharmingen, USA: PE-CY7 anti-human CD14 (clone: M5E2), PE-CF594 anti-human CD16 (clone:3G8), BV510 anti-human CD11b (clone: ICRF44), Percpcy5 anti-human CD11C (clone:F10/21A3), PE anti-human CD80 (clone: L307.4), FITC anti-human CD206 (clone: 19.2 (RUO), BV421 anti-human CD68 (clone:Y1/82A), BV711 anti-human CD141 (clone: 1A4), BV711 anti-human CD4 (clone:SK3), BV510 anti-human CD8 (clone:SK1), FITC anti-human CD3 (clone: HIT3a (RUO), BV421 anti-human CD25 (clone:M-A251), PE-CY7 anti-human; from Invitrogen USA: FOXP3 (clone:236A/E7), PE-CY5 anti-human CD69 (clone: CH/4), PE anti-human CDB220 (clone:RA3-6B2), PE anti-human TNF-α (clone: MAB11), BV421 anti-human IL-1β (clone:AS10) and FITC anti-human IL-6 (clone: MQ2-13A5 (RUO); from eBioscience: APC anti-human CD163 (clone: GHI/61); from R & D System: APC anti-human IFN-γ (clone: 25723) (human troponin-T (TNNT1) enzyme-linked immunosorbent assay (ELISA) kit catalog no. EHTNNT1 and human NPPB (BNP) ELISA kit catalog no. EHNPPB). Unless stated otherwise, these were used per the manufacturer’s recommendation.

### Experimental murine models

BALB/cJ (Jackson Laboratory: JAX: 000651) were purchased from The Jackson Laboratories. Mice of both genders at 10 weeks of age were utilized for experiments. Both male and female mice were randomly allocated to experimental groups. That said, a similar distribution of males and females between the experimental groups was always ensured. All the mice employed in this research were kept in a pathogen-free animal facility at the Bevill Biomedical Research Building, University of Alabama at Birmingham, Alamaba. They were handled in strict adherence to IACUC-approved protocol (IACUC no. 23012) and to the Guidelines for Animal Experiments established by the University of Alabama at Birmingham, Alabama, USA.

### Murine antibodies and reagents

The following antibodies and reagents were employed in the study: from BD Pharmingen, USA: APC-Cy7 anti-mouse CD45 (clone: 30-F11), PE-Cy7 anti-mouse CD3e (clone: 145-2C11), FITC anti-mouse CD4 (clone: RM4-5), PE-CF594 anti-mouse CD4 (clone: RM4-5), PE-CF594 anti-mouse TCR β chain (clone: H57-597), PerCP-Cy5.5 anti-mouse CD8a (clone: 53-6.7), BV421 anti-mouse IFN-γ (clone: XMG1.2), PE anti-mouse IL-4 (clone: 11B11), Alexa Fluor 700 anti-mouse IL-17A (clone: TC11-18H10), Alexa Fluor 647 anti-mouse IL-9 (clone: D9302C12), PE-Cy7 Anti-CD11b (clone: M1/70), FITC anti-mouse Ly-6C (clone: AL-21), BV421 anti-mouse F4/80 (clone: T45-2342), BV510 Rat anti-mouse Mer (clone: 108928), PE anti-mouse Ly-6G (clone: 1A8), APC anti-mouse Ly-6G and Ly-6C (clone: RB6-8C5), PE anti-mouse TNF (clone: MP6-XT22), Alexa Fluor 488 anti-mouse IL-6 (clone: MP5-20F3), BV421 anti-mouse CD25 (clone: PC61), purified rat anti-mouse CD16/CD32 (Mouse BD Fc Block), BV421 anti-mouse CD31 (clone: 390); From eBioscience, USA: APC IL-1β anti-mouse (clone: NJTEN3), Alexa Fluor 700 anti-mouse/Human Ki-67 (clone: SolA15), PerCP-Cyanine5.5 anti-mouse CD11c (clone: N418), PE anti-mouse CXCL-9 (clone: MIG-2F5.5), PE anti-mouse CD284 (TLR4) (clone: UT41), PE-Cyanine7 anti-mouse CD140a (PDGFRA) (clone: APA5), APC anti-mouse CD206 (clone: MR6F3), Alexa Fluor 700 anti-mouse MHC class II (I-A/I-E) (clone: M5/114.15.2); from R & D Systems: Alexa Fluor 647 anti-mouse CCL-2/JE/MCP-1 (clone: 123616R), Alexa Fluor 750 anti-mouse CCR2 (clone: 475301), Alexa Fluor 405 NLRP3/NALP3 anti-mouse/human (clone: 768319); from Miltenyi Biotec: APC Feeder Cells Antibody, anti-mouse, (clone: mEF-SK4). Additional reagents: ACTA2/smooth muscle actin rabbit anti-human, mouse, polyclonal, Proteintech, IgG (H + L) cross-adsorbed F(ab’)2-Goat anti-rabbit, PE, Invitrogen S100A8/A9 anti-mouse/human (clone: MRP8 7C12/4), Novus Biologicals, TNF-α mouse uncoated ELISA Kit (catalog no. 88-7324-22); from Invitrogen, IL-6 mouse uncoated ELISA Kit (catalog no. 88-7064-22) from Invitrogen, IL-1β mouse uncoated ELISA kit (catalog no. 88-7013-88) from Invitrogen. Unless stated otherwise, these were used per the manufacturer’s recommendation.

### Leukocyte isolation from cardiac tissue

Mice hearts underwent perfusion with phosphate-buffered saline (PBS), followed by collection and mincing. These minced heart tissues were then immersed in a digestion solution prepared in RPMI 1640 (Roswell Park Memorial Institute medium), which included collagenase I (catalog no. C0130, 1 mg/ml), collagenase XI (catalog no. C7657, 0.1 mg/ml), hyaluronidase (catalog no. H3506, 0.1 mg/ml) and DNase I (Sigma, catalog no. D4527, 1 μl/ml). The tissue was digested in a water bath at 37 °C with gentle agitation for 1 h, and the digested material was passed through 70-μm strainers. To stop the digestion process, RPMI 1640 media containing 5% fetal bovine serum (FBS) was added, and the mixture was then centrifuged at 1,300 rpm for 10 min at 4 °C to collect the leukocytes, which had settled at the bottom. The isolated cells were suspended in complete media (RPMI 1640 with 10% FBS), counted and utilized for subsequent flow cytometry staining.

### Flow cytometry analysis

For the processing of human samples, PBMCs obtained from participants’ blood were suspended in RPMI 1640 (Gibco, Invitrogen) supplementation with 10% human serum, 2 mM L-glutamine and 10 μg/ml penicillin–streptomycin and incubated for 30 min at the ice. The PBMCs were stained with fluorochrome-tagged antibodies and incubated for 30 min on ice. The cells were washed twice with PBS and the fluorescence intensity of fluorochrome-labeled cells was measured by flow cytometry (BD LSR-II) (UAB Flow Cytometry Core Facility). FACS Diva was used for acquiring the cells and final data analysis was performed using FlowJo (Tree Star). Mice hearts were subjected to digestion to isolate leukocytes, which were then quantified. Spleens from mice were isolated and macerated in RPMI 1640 containing 10% FBS to create a single-cell suspension. Red blood cells) were lysed using red blood cells lysis buffer (catalog no. 118-156-101, Quality Biological), followed by incubation at room temperature for 1 min and subsequent washing with RPMI 1640 supplemented with 10% FBS. The cells were counted, and 0.5–1 × 10^6^ cells were used for surface staining after Fc receptor blocking (1 μg/ml) in 3% FBS for 30 min on ice. For intracellular staining, 0.5–1 × 10^6^ cells were cultured in 96-well plates (Nunc) and incubated for 5 h in RPMI media containing Phorbol 12-myristate 13-acetate or PMA (Phorbol 12-myristate 13-acetate) (0.1 mg/ml, Sigma), ionomycin (1 mg/ml, Sigma) and a 1:1,000 dilution of Golgistop/Golgiplug (BD Biosciences). Following stimulation, the cells were first surface stained for 30 min on ice. After washing with PBS, cells were fixed and permeabilized using the BD cytofix/cytoperm kit (BD Biosciences) for 30 min at 4 °C, followed by washing with the BD perm wash kit (BD Biosciences) and then stained with intracellular fluorescently labeled antibodies. The fluorescence intensity of fluorochrome-labeled cells was measured using flow cytometry (BD LSR-II), and sorting, as needed, was performed with the BD FACS Aria III cytometer at the UAB Flow Cytometry Core Facility. FACS Diva was utilized for cell acquisition, and the final data analysis was conducted using FlowJo (Tree Star).

### ELISA for quantifying proinflammatory cytokines (IL-1β, IL-6 and TNF-α)

Sera were obtained from experimental mice. According to the manufacturer’s instructions, the concentrations of IL-1β, IL-6 and TNF-α and S100A8/9 heterodimer (R & D system, catalog no. DY8596-05) were assessed in the sera using ELISA kits (catalog no. 88-7013-88, 88-7064-22 and 88-7324-22). The concentrations of human HF markers (troponin-T and BNP) were assessed in the sera using ELISA kits (human troponin-T (TNNT1) ELISA kit catalog no. EHTNNT1 and human NPPB (BNP) ELISA kit catalog no. EHNPPB).

### Multiplex cytokine/chemokine assay

The concentrations of cytokines and chemokines in human blood sera were assessed using outsourced services from Eve Technologies.

### Insulin, glucose and cholesterol assays

Serum glucose and cholesterol concentration levels were detected using a mouse Insulin assay kit supplied by Crystal Chemical (catalog no. 90080), a glucose assay kit supplied by Crystal Chemical (catalog no. 81692), and a cholesterol reagent with a standard kit (RAICHWM reference no. R80035) according to the manufacturer’s instructions.

### Transthoracic echocardiography

Echocardiography was conducted using the Vevo 3100 high-resolution ultrasound imaging system equipped with an MX400 transducer (Visual Sonics). During the process of the echocardiogram, mice were anesthetized with 1.5–2% isoflurane and positioned on a temperature-controlled platform in a supine orientation. After removing the hair, a layer of warmed ultrasound transmission gel was applied to the chest area. M-mode echocardiography was performed in the parasternal short-axis view, precisely at the level of the greatest left ventricular end-diastolic dimension. Various cavity dimensions and wall thicknesses were quantified using the M-mode interrogation. Parameters such as ejection fraction, fractional shortening and other relevant metrics were calculated based on the short-axis view utilizing the Vevo 3100 software. Mice with heart rates below 400 beats per minute were excluded from the analysis.

### Tissue processing, histological analysis and immunofluorescence assay

Animals were euthanized and hearts were collected, fixed in formalin and embedded in paraffin using established protocols as described in previous publications^18^. To assess collagen deposition, tissue sections were stained with Masson’s trichrome (Sigma-Aldrich). A quantification of fibrosis was carried out on Masson’s trichrome-stained tissue sections with the assistance of NIH ImageJ software. CD45 immunostaining was conducted following well-established protocols outlined in prior publications^18^. Fluorescence imaging was performed using a KEYENCE BZ-X800

### Infection, antibiotic and hydrocortisone and paquinimod treatment

The experimental design and timeline of treatments and readouts are depicted in Fig. 3a. In brief, *Spn* was grown to exponential phase in Todd-Hewitt Broth at 37 °C in 5% CO_2_ to an OD_621_ of 0.4. Pneumococci were diluted 1:1,000 in sterile PBS, and the mice were infected intraperitoneally with 100 µl of the suspension using a tuberculin syringe. Each mouse was infected with ~1 × 10^4^ CFU of *Spn*, resulting in bacteremia, similar to what occurs during IPD. Starting at 30 h post infection and every 12 h after that for 3 days, infected mice were administered 50 mg/kg of filter-sterilized ampicillin dissolved in PBS in a volume of 200 μl intraperitoneally. Following this, mice were given water with filter-sterilized ampicillin (1 mg/ml) ad libitum for 4 days. Blood was collected on day 3 and 7 from the tail vein to assess antibiotic killing of the bacteria via plating and culture on blood agar plates. In mice that were treated with hydrocortisone, starting at 30 h post infection, and in addition to ampicillin, 5 mg/kg body weight of hydrocortisone suspended in 100 µl of PBS was administered intraperitoneally daily in beginning for 1 week, and then, 1.5 mg/kg was given for further 2 weeks. For mice that received paquinimod, 3.75 mg/kg paquinimod was administered ad libitum in drinking water for 3 weeks as used previously^8^.

### Immunoblotting of NFκB and phosphorylated NFκB

The extract was prepared from whole tissue by homogenization in 1× RIPA buffer (Cell Signaling, #9806, 10×) supplemented with 1× protease/phosphatase inhibitor cocktail (#5872, 100×). Tissue homogenization was carried out using a bead mill tissue homogenizer for 3 min at 4 °C, followed by centrifugation to remove insoluble cell debris. For subcellular fractionation, ice-cold cytoplasmic extraction buffer containing protease inhibitors (Thermo Scientific, #87790) was added to the tissue, which was then homogenized for 3 min at 4 °C using a bead mill homogenizer. The homogenized tissue was transferred into a Pierce tissue strainer and centrifuged at 500*g* for 5 min. The supernatant (cytoplasmic extract) was immediately transferred into a clean, prechilled tube on ice. The remaining pellet was resuspended in ice-cold nuclear extraction buffer containing protease inhibitors (Thermo Scientific, #87790). Following 15 s of vortexing and a 30-min incubation at 4 °C, the sample was centrifuged at 5,000*g* for 5 min. The resulting supernatant, containing the soluble nuclear extract, was collected into a clean, prechilled tube on ice. Protein concentration in the extracts was determined using a bicinchoninic acid (BCA) assay (Sigma) according to the manufacturer’s instructions. Tissue extracts (40 µg) were resolved by 12% SDS–polyacrylamide gel electrophoresis, followed by electrophoretic transfer to nitrocellulose membranes. Immunoblotting was performed using standard protocols. Membranes were blocked with 5% skimmed milk in 1% TBS–Tween and incubated with primary antibodies: rabbit anti-NFκB mAb (1:1,000) (p65, Cell Signaling, #4764S), rabbit anti-pNFκB mAb (1:1,000) (Cell Signaling, #3033T), mouse anti-GAPDH mAb (1:3,000) (MilliporeSigma, #CB1001-500UG) and rabbit anti-histone H3 mAb (1:1,000) (Cell Signaling, #4499). For detection, membranes were incubated with HRP-conjugated secondary antibodies: goat anti-mouse IgG (1:5,000) (Fisher, #0B1030-05) and goat anti-rabbit IgG (1:5,000) (Fisher, #NC0977124). The blots were visualized using enhanced chemiluminescence.

### Statistical analyses

We conducted our analyses using GraphPad Prism (version 9.3.1). The data were subjected to statistical analysis based on the following criteria: we employed an unpaired, two-tailed Student *t*-test for comparisons involving two groups. When dealing with multiple groups, we initially performed a one-way analysis of variance (ANOVA). Post hoc analysis following the Kruskal–Wallis test was carried out using the Dunn method. We considered a significance level of less than 0.05 (*P* < 0.05) statistically significant.

## Results

### IPD survivors manifest impaired HF markers and unresolved Inflammation through elevated levels of myeloid cells

**Fig. 1:**
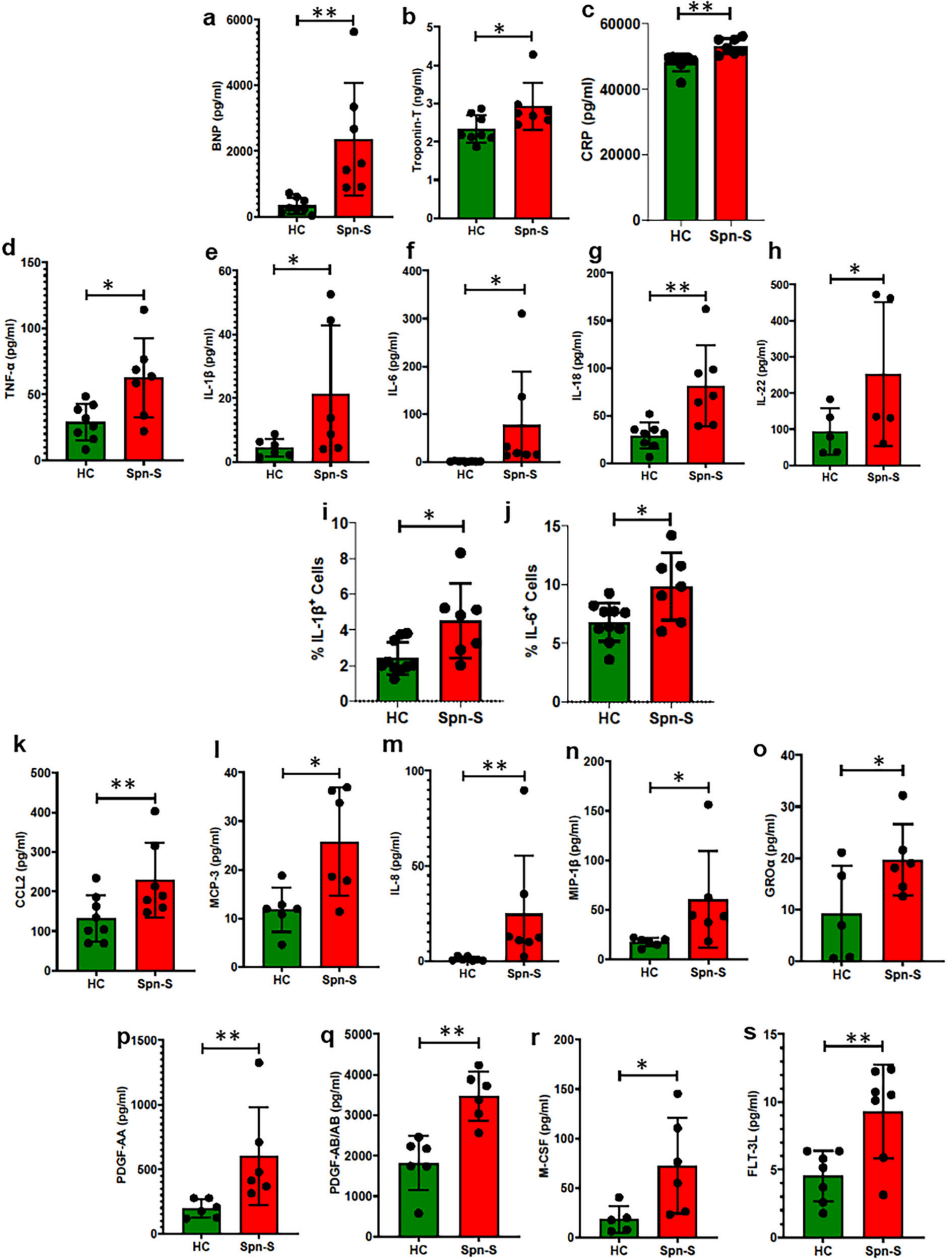
Post-*Spn*-infection-survived patients lead impaired HF markers and chronic inflammation. **a** BNP assays were performed following the protocol provided by the kit manufacturer (catalog no. EHNPPB) using serum samples collected from healthy and pneumonia-survived patients (*Spn*-S), healthy and *Spn*-survived (*N* = 7–8). **b** Troponin-T assays were performed following the protocol provided by the kit manufacturer (catalog no. EHTNNT1) using serum samples collected from healthy and pneumonia-survived patients, healthy and *Spn*-survived (*N* = 7–8). **c** CRP assays were performed following the protocol provided by the kit manufacturer (catalog no. KHA0031) using serum samples collected from healthy and pneumonia-survived patients, healthy and *Spn*-survived (*N* = 7–8). **d**–**h** Cytokines were assessed by Eve Technologies with the provided serum samples collected from healthy and *Spn*-survived patients: TNF-α (*N* = 7–8) (**d**), IL-1β (*N* = 6) (**e**), IL-6 (*N* = 7–8) (**f**), IL-18 (*N* = 7–8) (**g**), IL-22 (*N* = 5) (**h**). PBMCs were isolated from both healthy individuals and those who survived pneumonia. **i**,**j** Subsequently, flow cytometry analysis was conducted to determine the percentage of various subpopulations: the percentage of IL-1β-producing cells (*N* = 7–10) (**i**) and the percentage of IL-6-producing cells (*N* = 7–10) (**j**). **k**–**s**, Chemokines were assessed by Eve Technologies with the provided serum samples collected from healthy and *Spn*-survived patients: CCL-2 (*N* = 7–8) (**k**), MCP-3 (*N* = 6) (**l**), IL-8 (*N* = 7) (**m**), MIP-1β (*N* = 6) (**n**), GRO-α (*N* = 5-6) (**o**), PDGF-AA (*N* = 6) (**p**), PDGF-AB/AB (*N* = 6) (**q**), M-CSF (*N* = 5–6) (**r**), FLT-3L (*N* = 7) (**s**). *P < 0.05, **P < 0.01. The data are presented as mean ± standard deviation, and the significance of the data was determined using a two-tailed Mann–Whitney *U* test.

**Fig. 2:**
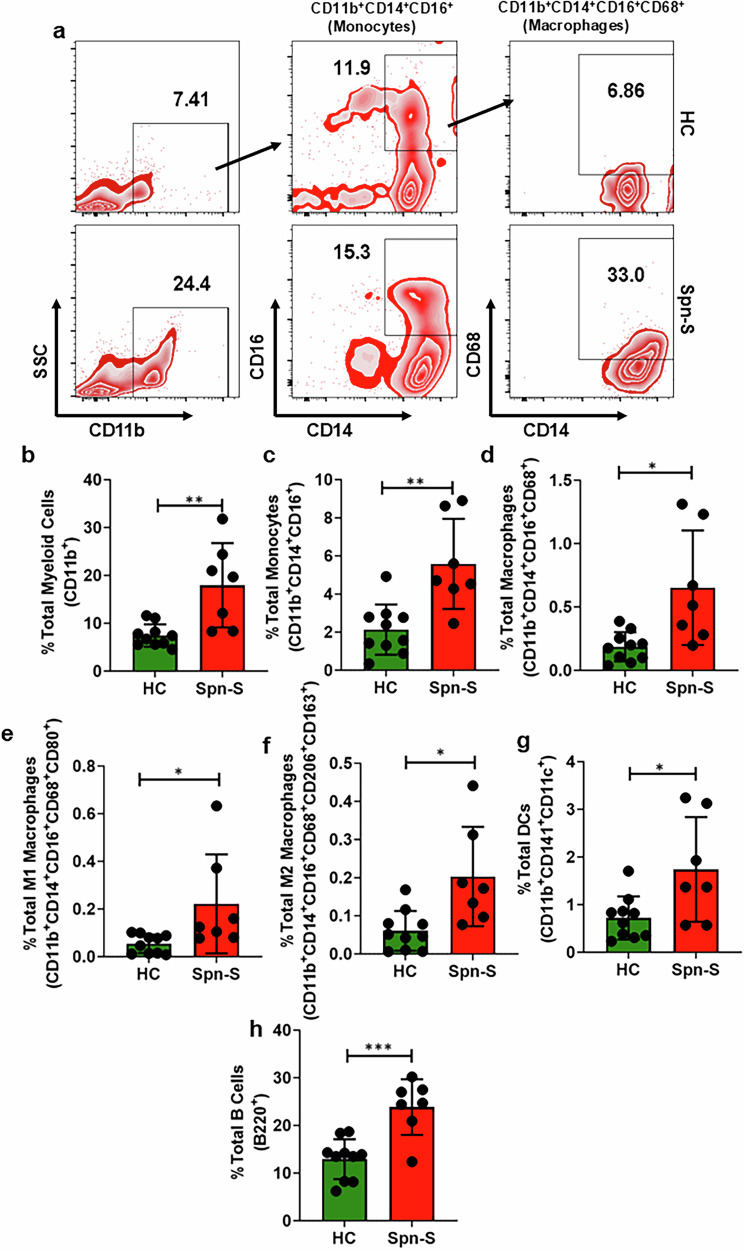
Postinfection survivors exhibit unresolved inflammation through elevated innate immune cell (myeloid cell) frequencies. **a**–**h** Representative flow plot demonstrating myeloid cells, monocytes and macrophages (**a**) gating the percentage of total myeloid cells (*N* = 7–10) (**b**), percentage of total monocytes (*N* = 7–10) (**c**), percentage of total macrophages (*N* = 7–10) (**d**), percentage of total M1 macrophages (*N* = 7–10) (**e**), percentage of total M2 macrophages (*N* = 7–10) (**f**), percentage of total dendritic cells (DCs) (*N* = 7-10) (**g**) and percentage of total B cells (*N* = 7–10) (**h**). The PBMCs were isolated from both healthy individuals and those who survived pneumonia. Subsequently, flow cytometry analysis was conducted to determine the percentage of various subpopulations *P < 0.05, **P < 0.01. The data are presented as mean ± standard deviation, and the significance of the data was determined using a two-tailed Mann–Whitney *U* test.

First, to assess ongoing cardiac stress/damage in patients hospitalized for severe *Spn* infection who had earlier received antibiotic treatment to eradicate the bacterium, we measured HF markers in their blood serum 5 days after admission. As expected, elevated levels of plasma N-terminal probrain natriuretic peptide (NT-proBNP) and troponin-T were detected in convalescent patients; albeit levels were considerably higher from BNP, which is more indicative of HF than acute damage (Fig. 1a, b). C-reactive protein (CRP) is recognized as an inflammation marker and has been identified as a potential biomarker for HF^26,27^. However, it is not specific to HF and can be elevated in various inflammatory conditions^28^. However, it is often used alongside other biomarkers such as BNP and troponin-T to strongly correlate with occurrence of HF^29^. Along with BNP and troponin-T, elevated CRP levels in *Spn*-survived patients supported our hypothesis that IPD survivors may exhibit HF manifestations (Fig. 1c) Inflammation is typically tightly regulated, and we sought to investigate whether systemic inflammation might also be present during this resolution stage. We observed elevated levels of TNF-α, IL-1β, IL-6, IL-18 and IL-22 in patients with *Spn* compared with healthy individuals (Fig. 1d–h). Flow cytometry analyses conducted with the patient’s PBMCs corroborated a sustained inflammatory state with a greater number of IL-1β and IL-6-producing cells among those admitted for *Spn* disease (Fig. 1I,j). We also observed elevated levels of the chemokines CCL-2, MCP-3, IL-9, MIP-1, GROa, PDGF-AA and PDGF-AB/AB (Fig. 1k–q). Notably, elevated levels of myeloid cell proliferating factors, such as macrophage colony-stimulating factor (M-CSF) and FMS-like tyrosine kinase 3 ligands (FLT-3L) were also observed. The latter finding suggests that cells originating from the myeloid lineage were undergoing proliferation (Fig. 1r,s). Along such lines, and upon immunophenotyping of isolated PBMCs, we observed considerably enhanced frequencies of monocytes, macrophages, M1 and M2 macrophages and dendritic cells (Fig. 2a–g). Flow cytometry analysis revealed a predominance of CD14⁺CD16^−^ classical monocytes within the PBMCs, indicative of a proinflammatory immune profile (Fig. 2a). This shift is consistent with early immune activation in the context of infection or disease^30–32^. CD68 is commonly used as a pan-monocyte/macrophage marker, but its expression is low in circulating monocytes and higher in tissue macrophages^33,34^. Accordingly, we observed weak CD68 expression in blood monocytes, consistent with previous reports and explaining its limited detection in our panel^33,34^ (Fig. 2a). In addition, we observed a higher frequency of B cells in IPD survivors compared with healthy controls (Fig. 2h). Together, these observations are evidence of both heart damage and unresolved inflammation in *Spn* pneumonia survivors.

### IPD leads to cardiac dysfunction and chronic inflammation in the *Spn*-survived animal model

**Fig. 3:**
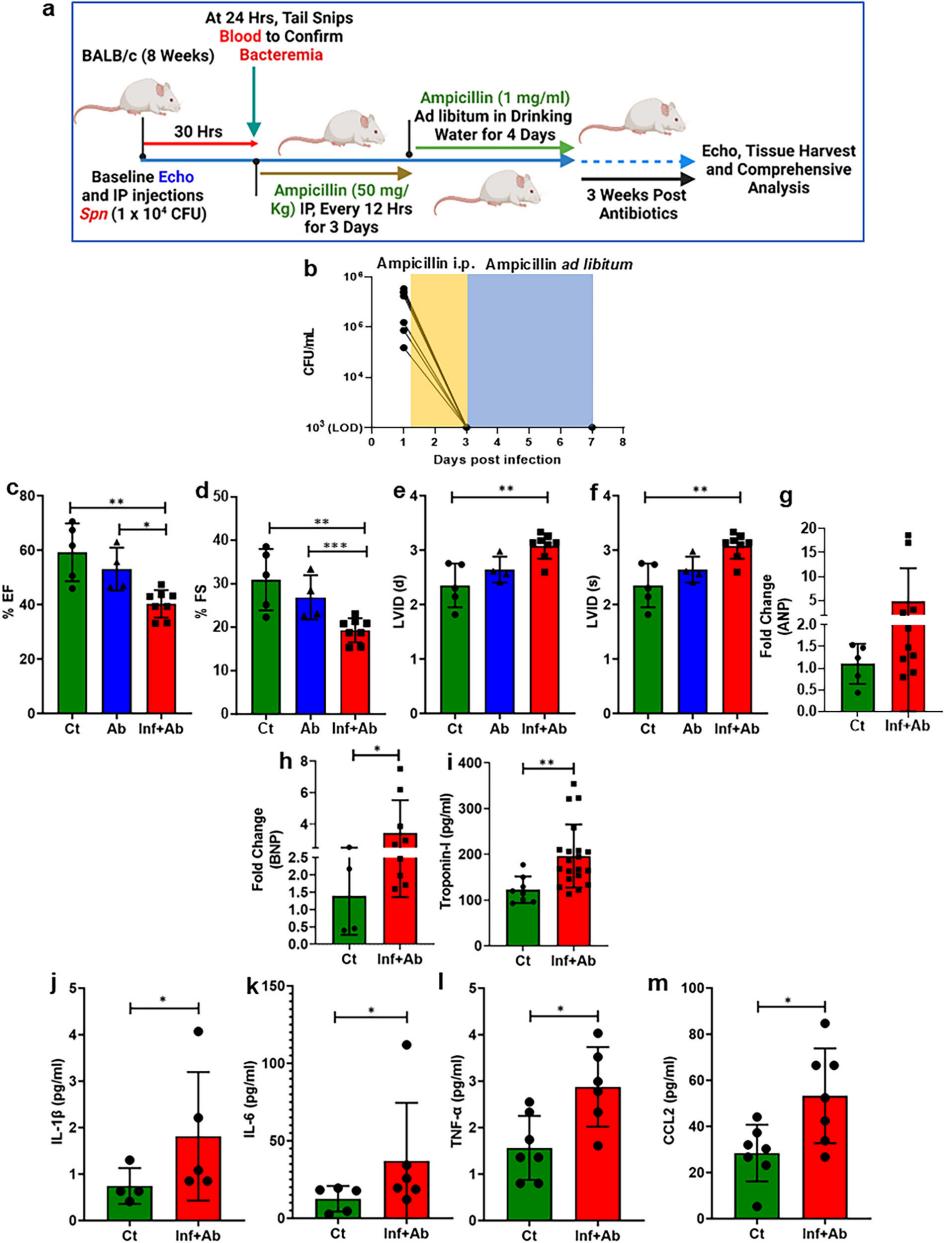
Post-*Spn-*infection-survived mouse model shows cardiac dysfunction and persistent Inflammation. **a** A schematic diagram illustrating the approach to demonstrate models for studying cardiac functional impairment 3 weeks post clearance of bacterial burden. Ct, control (naive animals); Inf+Ab, infected then antibiotic-treated animals. **b** The graph represents CFU before and post ampicillin treatment (*N* = 7). **c** Ejection fraction (EF) (*N* = 5–8). **d** Fraction shortening (FS) (*N* = 5–8). **e** Left ventricular internal dimension (LVID) diastolic (*N* = 5–8). **f** LVID systolic (*N* = 5–8). **g** ANP fold change measured by qRT–PCR (5–10). **h** BNP fold change measured by qRT–PCR (4–9). **i** Serum troponin-I measured by ELISA (*N* = 8–20). **j** Serum IL-1β measured by ELISA (*N* = 4–5). **k** Serum IL-6 measured by ELISA (*N* = 5–6). **l** Serum TNF-α measured by ELISA (*N* = 6–7). **m** Serum CCL-2 concentration (*N* = 7). *P < 0.05, **P < 0.01. The data are presented as mean ± standard deviation, and the significance of the data was determined using a two-tailed Mann–Whitney *U* test.

Individuals hospitalized for pneumonia typically have multiple underlying morbidities, including cardiovascular disease, and experience some form of chronic inflammation^1,2^. To rule out these confounders and extend our analyses of post-CAP inflammation beyond 5 days, we investigated the effect of severe *Spn* infection, that is, IPD, on cardiac remodeling and function 3 weeks following successful antimicrobial treatment for the infection. The experimental design, including the timeline of treatments and readouts, is depicted in Fig. 3a and described in the method section. CFU analysis in these animals confirmed complete bacterial clearance following the 1 week ampicillin treatment regimen (Fig. 3b and Supplementary Table 2). Foremost, echocardiography of mice revealed marked reductions in ejection fraction and fractional shortening as well as changes in left ventricular internal diameter at both end diastole and end systole in the *Spn*-infected rescued group versus both control groups (Fig. 3c–f). Similar to our results with human samples, we also observed increased serum levels of the cardiac damage markers ANP, BNP and troponin-I (Fig. 3g–i). However, despite elevated levels of troponin-I at 48 h post infection and during the convalescent period, no notable cardiomyocyte death was observed 3 weeks after the ampicillin treatment Fig. 3i and Supplementary Fig. 1a,b). Moreover, no signs of hypertrophy were detected during the convalescent period in IPD-survived animals (Supplementary Fig. 1c). Increased serum levels of IL-1β, IL-6 and TNF-α in *Spn*-survived animals were also detected (Fig. 3j–l). Finally, we also observed elevated levels of serum CCL-2, suggesting myeloid cell infiltration into tissues was also occurring (Fig. 3m). Altogether, these results add to the body of evidence that severe *Spn* infection leads to adverse cardiac remodeling and dysfunction that persists beyond the actual infection. They also indicate that persistent chronic inflammation is present and, therefore, may be a critical contributor to this phenomenon.

### Persistence activation of S100A8/9-TLR4-NLRP3-NF-ƙβ signaling in the hearts of convalescent mice

**Fig. 4:**
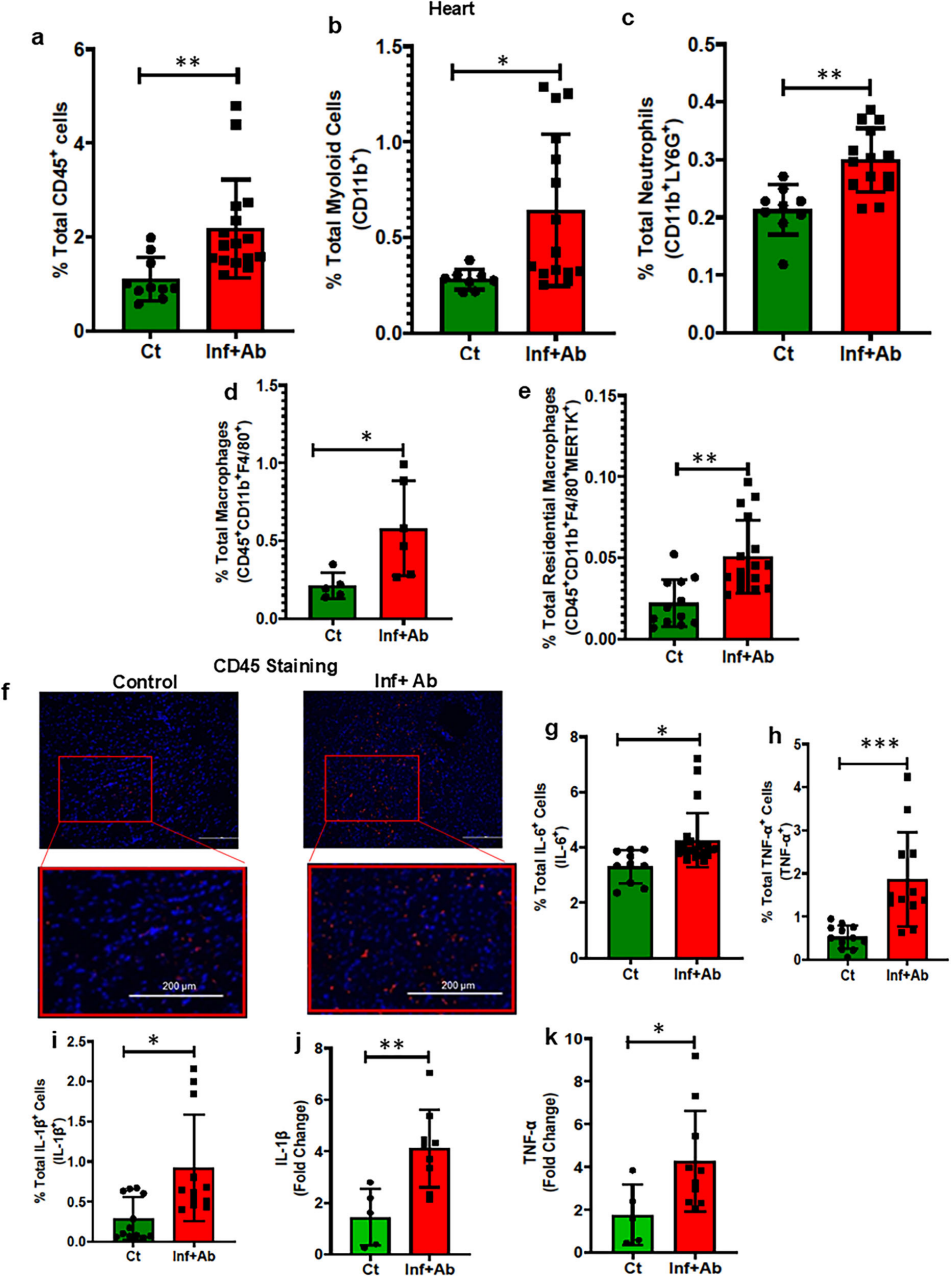

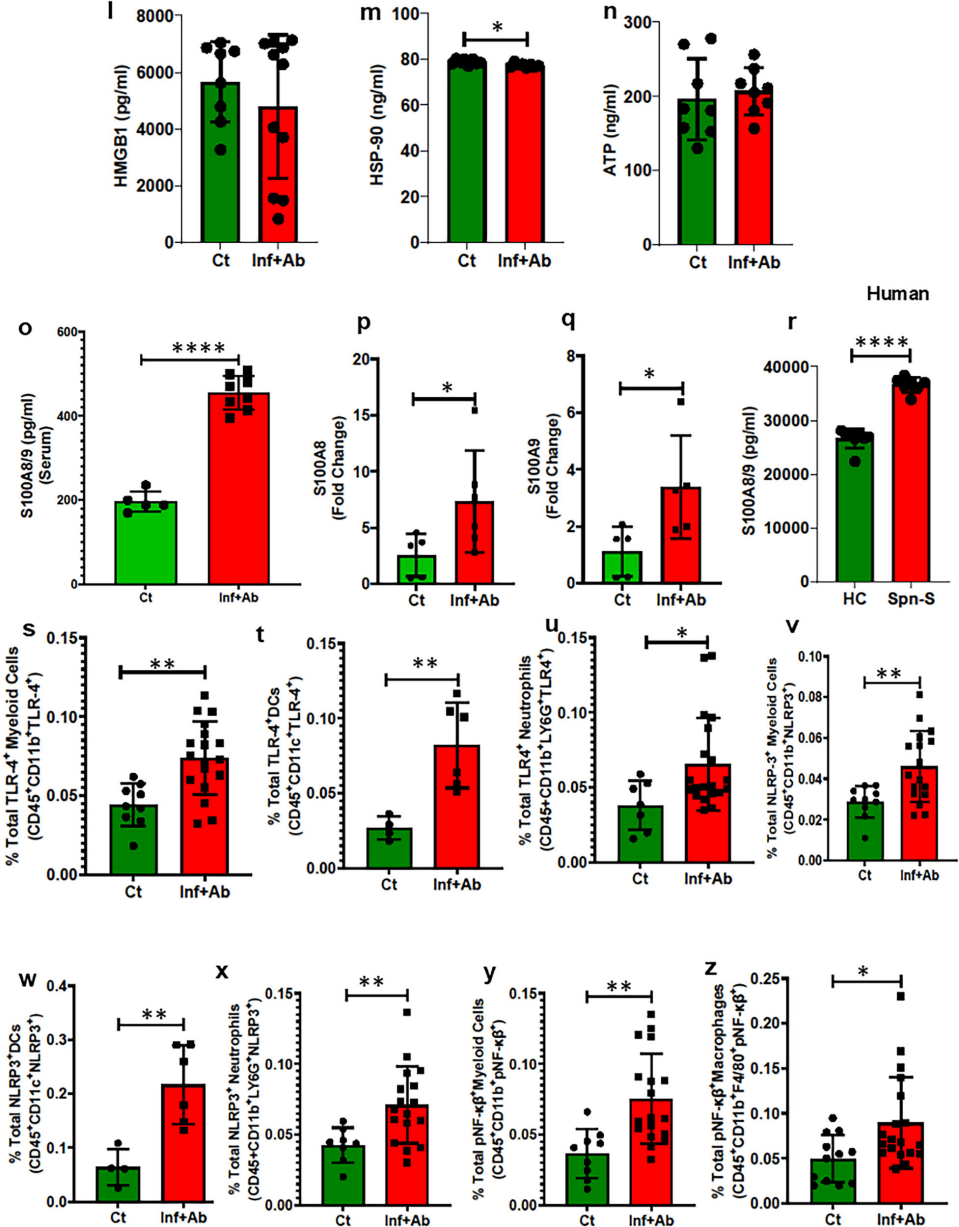
Post-*Spn-*infection-survived mice show cardiac chronic inflammation via NF-ƙB activation in myeloid cells through the S100A8/9-TLR4-NLRP3-NF-ƙB axis. **a**–**f** Total percentages of CD45^+^ cells (*N* = 10–16) (**a**), CD11b^+^ myeloid cells (*N* = 10–18) (**b**), CD11b^+^LY6G^+^ neutrophils (*N* = 9–18) (**c**), CD45^+^CD11b^+^F4/80^+^ macrophages (*N* = 5–6) (**d**), CD45^+^CD11b^+^F4/80^+^MERTK^+^ residential macrophages (*N* = 12–18) (**e**), IHC showing CD45^+^ leukocytes (200 μm scale) (**f**). **g**–**i** Total percentages of IL-6-producing cells (*N* = 10–16) (**g**), TNF-α-producing cells (*N* = 12–16) (**h**) and IL-1β-producing cells (*N* = 12–16) (**i**). The percentage of IL-6-, TNF-α- and IL-1β-producing cells were measured across all the cells including Immune and nonimmune cells. **j**, IL-1β fold change measured by qRT–PCR (*N* = 5–9). **k** TNF-α fold change measured by qRT–PCR (*N* = 5–9). **l** Mouse High Mobility Group Protein B1(HMGB1) assays were performed following the protocol provided by the kit manufacturer (catalog no. EEL102) using serum samples collected from control and pneumonia-survived animals, (*N* = 8–10). **m** Mouse ATP assays were performed following the protocol provided by the kit manufacturer (catalog no. MBS267352) using serum samples collected from control and pneumonia-survived animals (*N* = 8). **n** Mouse HSP90 assays were performed following the protocol provided by the kit manufacturer (catalog no. NBP2-76449) using serum samples collected from control and pneumonia-survived animals (*N* = 8). **o**, The concentration of S100A8/9 serum measured by ELISA (*N* = 5–8). **p** S100A8 fold change measured by qRT–PCR (*N* = 5–6). **q** S100A9 fold change measured by qRT–PCR (*N* = 5). **r** Human S100A8/9 assays were performed following the protocol provided by the kit manufacturer (catalog no. DY8226-05) using serum samples collected from healthy and pneumonia-survived patients (*N* = 7–8). **s**–**z** The total percentages of TLR4^+^ myeloid cells (*N* = 9–18) (**s**), TLR4^+^ dendritic cells (DCs) (*N* = 4–6) (**t**), TLR4^+^ neutrophils (*N* = 7–18) (**u**), NLRP3^+^ myeloid cells (*N* = 10–18) (**v**), NLRP3^+^ DCs (*N* = 4–6) (**w**), NLRP3^+^ neutrophils (*N* = 8–18) (**x**), pNF-ƙβ^+^ myeloid cells (*N* = 9–18) (**y**), pNF-ƙβ^+^ macrophages (*N* = 12–18) (**z**). The left ventricles (LV) of the hearts of control and infected, then antibiotic-treated, animals were digested to create a single-cell suspension. Subsequently, these cells were stained with a panel of antibodies, and flow cytometry was performed. *P < 0.05, **P < 0.01, ***P < 0.001, ****P < 0.0001. The data are presented as mean ± standard deviation, and the significance of the data was determined using a two-tailed Mann–Whitney *U* test.

To gain better insight as to whether immunological processes were contributing to *Spn*-mediated cardiac remodeling and dysfunction post infection, infiltration of immune cells into the heart and proinflammatory signaling were determined. The experimental design, treatment and readout timelines are depicted in Fig. 3a. Flow cytometry analysis revealed an increased percentage of myeloid cells, including neutrophils and macrophages, in the hearts of *Spn*-survived mice versus healthy controls (Fig. 4a–e). Robust infiltration of CD45^+^ immune cells into the myocardium of *Spn*-survived hearts was also consistently observed via immunofluorescent microscopy (Fig. 4f). Intracellular staining for IL-6, TNF-α and IL-1β revealed a higher number of cells producing these proinflammatory cytokines in the hearts of *Spn*-rescued mice (Fig. 4g–i). Consistently, increased levels of gene expression corresponding to the IL-1β and TNF-α encoding genes were also observed (Fig. 4j, k).

Robust immune cell infiltration in the hearts of *Spn*-survived animals even 3-weeks after the clearance of bacteria, prompted us to screen for the sustained presence of DAMPs capable of activating innate immune cells and inducing chronic inflammation. Interestingly, we observed no comparable difference in HMGB1 and extracellular ATP levels between control and IPD-survived animals (Fig. 4l,m). Moreover, a diminished level of HSP90 in survived animals prompted the exclusion of these DAMPs from the study (Fig. 4n). By contrast, we observed elevated serum S100A8/A9 levels in *Spn*-survived animals (Fig. 4o). PCR analysis also demonstrated criticaly upregulated expression of S100A8 and S100A9 in isolated hearts of *Spn*-survived animals (Fig. 4p–q); which was consistent with the increased myocardial infiltration of myeloid cells. The elevated levels of serum S100A8/9 observed in *Spn-*surviving patients also indicated S100A8/9-mediated inflammation (Fig. 4r). To gain insight into mechanisms responsible for proinflammatory response, we analyzed whether IPD altered the classical S100A8/A9-TLR4-NLRP3-pNF-ƙβ- signaling axis in vivo. Indeed, IPD increased the number of TLR4 and NLRP3 expressing myeloid cells, dendritic cells and neutrophils in the heart (Fig. 4s–x), as well as myeloid cells and macrophages with activated NFkB (Fig. 4y,z).

### Inflammation post pneumonia is systemic

**Fig. 5:**
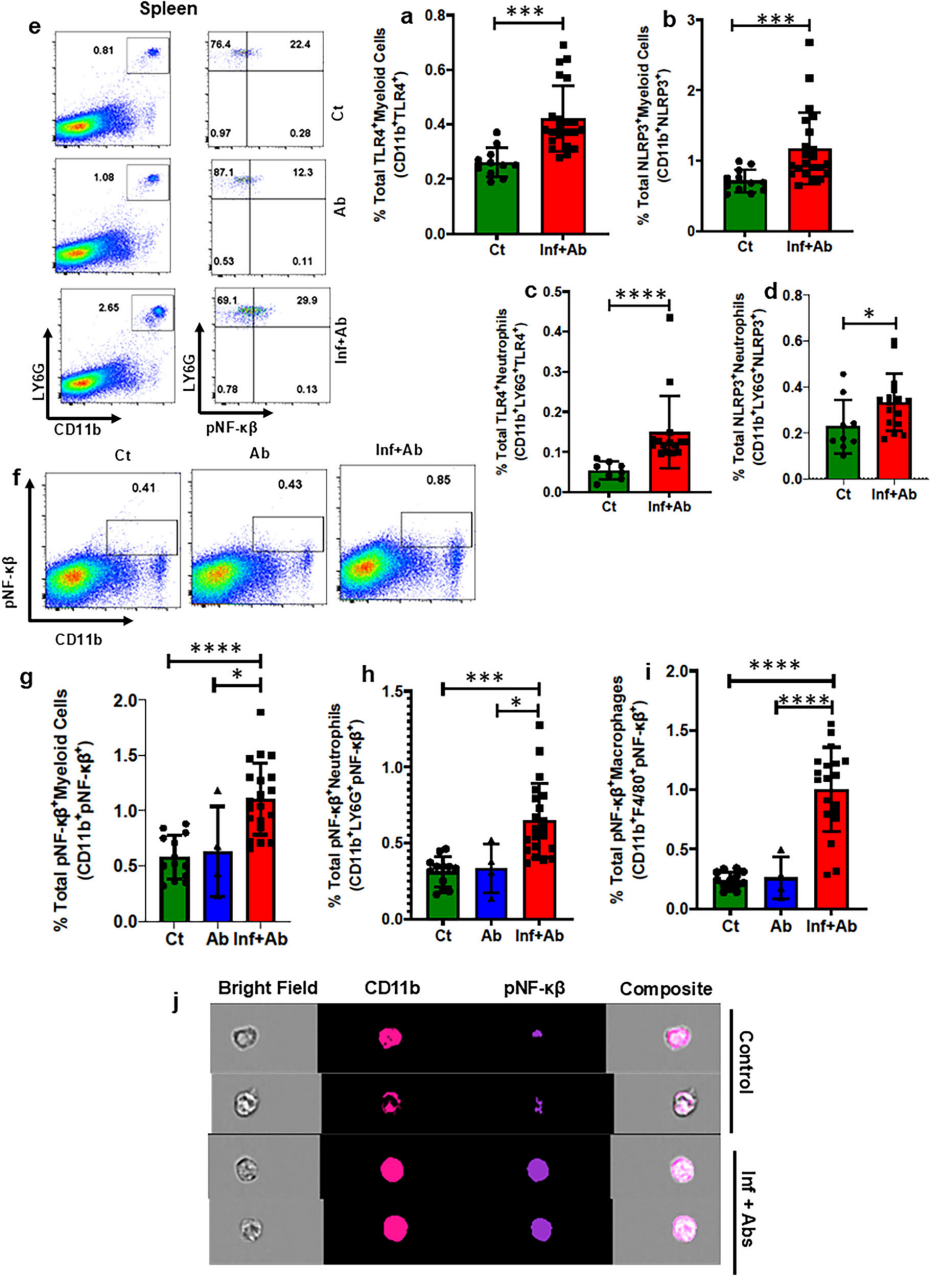

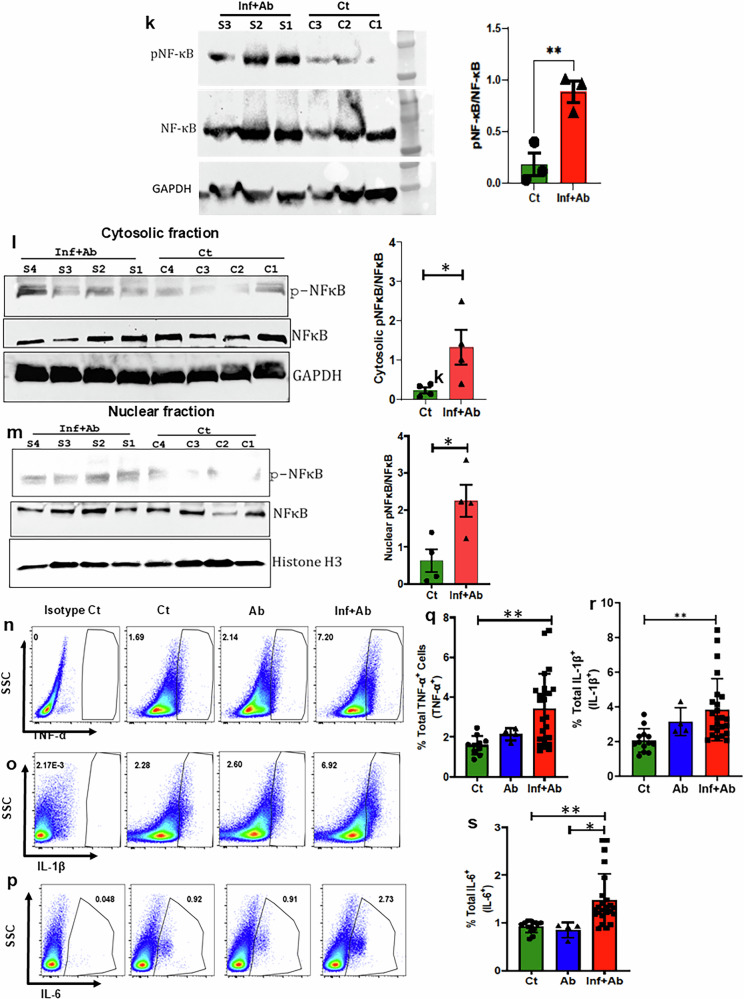
Post-*Spn*-infection-survived mice show systemic chronic inflammation via NF-ƙβ activation in myeloid cells through the S100A8/9-TLR4-NLRP3-NF-ƙβ axis. **a** The data represent a quantitation of the percent of total TLR4^+^ myeloid cells (*N* = 11–18). **b** The data represent the quantitation of the percent of total NLRP3^+^ myeloid cells (*N* = 11–18). **c** The data represent a quantitation of the percent of total TLR4^+^ neutrophils (*N* = 8–18). **d** The data represent a quantitation of the percent of total NLRP3^+^ neutrophils (*N* = 9–16). **e** A representative figure of flow cytometry showing gating strategy to measure pNF-ƙβ^+^ neutrophils from the spleen of all groups. **f** A representative figure of flow cytometry showing gating strategy to measure pNF-ƙβ^+^ myeloid cells from the spleen of all groups. **g** The data represent a quantitation of the percent of total pNF-ƙβ^+^ myeloid cells (*N* = 4–18). **h** The data represent a quantitation of the percent of total pNF-ƙβ^+^ neutrophils (*N* = 4–18). **i** The data represent a quantitation of the percent of total pNF-ƙβ^+^ macrophages (*N* = 4–18). **j** A representative figure of image stream showing pNF-ƙβ^+^ myeloid cells from the spleen of all groups. Whole heart tissue lysates, as well as cytosolic and nuclear fractions, were prepared from *Spn*-infected plus antibiotic-treated (Inf + Ab) and control mice. **k** Representative immunoblot images of phosphorylated NFκB (pNFκB), total NFκB and GAPDH (loading control) from whole heart tissue extracts, along with the corresponding pNFκB/NFκB quantification data for both groups (*n* = 3 per cohort). **l** Representative immunoblot images and quantification data of pNFκB and total NFκB in the cytosolic fraction of heart tissue (*n* = 4 per cohort), with GAPDH as a loading control for the cytosolic fraction. **m** Representative immunoblot images and quantification data of pNFκB and total NFκB in the nuclear fraction of heart tissue (*n* = 4 per cohort), with histone H3 as a loading control for the nuclear fraction. **n** A representative figure of flow cytometry showing gating strategy to measure TNF-α^+^ cells from the spleen of all groups. **o** A representative figure of flow cytometry showing gating strategy to measure IL-1β^+^ cells from the spleen of all groups. **p** A representative figure of flow cytometry showing gating strategy to measure IL-6^+^ cells from the spleen of all groups. **q** The data represent a quantitation of the percent of total TNF-α-producing cells (*N* = 4–18). **r** The data represent a quantitation of percent of total IL-6-producing cells (*N* = 4–18). **s** The data represent a quantitation of the percent of total IL-1β-producing cells (*N* = 4–18). *P < 0.05, **P < 0.01, ***P < 0.001, ****P < 0.0001. The data are presented as mean ± standard deviation, and the significance of the data was determined using a two-tailed Mann–Whitney *U* test when compared between two groups, whereas the significance is measured by one-way ANOVA when compared between three groups.

The spleen serves as a secondary lymphoid organ crucial for regulating systemic immune responses; therefore, the characterization of immune cells within the spleen is a good representative of systemic immune status. In the spleen of mice post infection, we noted an augmentation in the abundance of myeloid cell populations, encompassing total macrophages, M1/M2 ratio, dendritic cells and neutrophils (Supplementary Fig. 1d–o). Consistent with our results in the heart, the percentage of TLR4^+^ myeloid cells, NLRP3^+^ myeloid cells, TLR4^+^ neutrophils, NLRP3^+^ neutrophils, pNF-ƙβ^+^ myeloid cells, pNF-ƙβ^+^ neutrophils and pNF-ƙβ^+^ macrophages were all also increased in the spleen (Fig. 5a–i and Supplementary Fig. 2). Advanced image stream single-cell analysis further confirmed *Spn*-induced NLRP3-pNF-ƙβ activation in myeloid cells at a single-cell level (Fig. 5j). Western blot analysis using whole heart lysates supported the hypothesis that NF-ƙB remained activated during the convalescent period in IPD-survived animals (Fig. 5k). Furthermore, data from cytosolic and nuclear fractions of heart samples clearly demonstrate NF-ƙB translocation to the nucleus and thereby capable of driving sustained inflammatory activation and chronic inflammation in IPD-survived animals (Fig. 5l,m). Intracellular staining for cells producing proinflammatory cytokines provided additional support for activation of the S100A8/A9-TLR4-NLRP3-pNF-ƙβ proinflammatory axis (Fig. 5n–s and Supplementary Fig. 3). Thus, IPD results in a prolonged systemic inflammatory state. In addition, the inclusion of an antibiotic-treated control group revealed that NF-ƙβ activation was comparable with untreated controls (Fig. 5e–i), although a modest trend of upregulation in IL-1β (Fig. 5r) was observed in the antibiotic-treated control group, suggesting a limited but measurable impact of microbiota depletion on inflammatory responses.

### Proinflammatory T cells and antigen-specific T cells are present in human and mouse *Spn* survivors

**Fig. 6:**
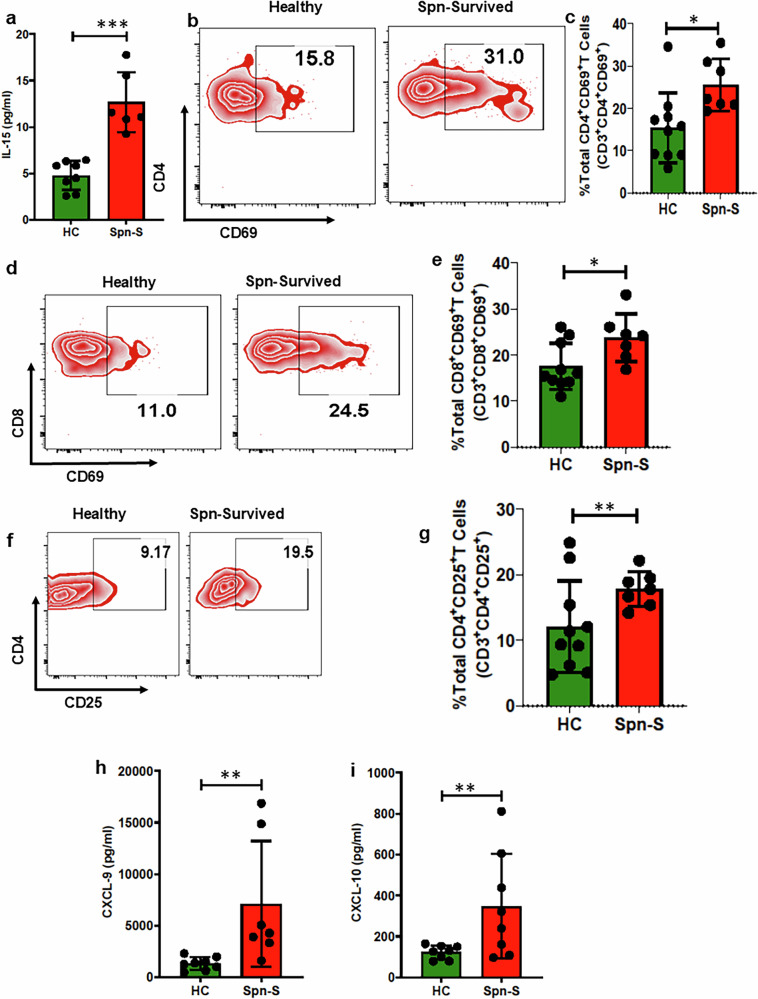

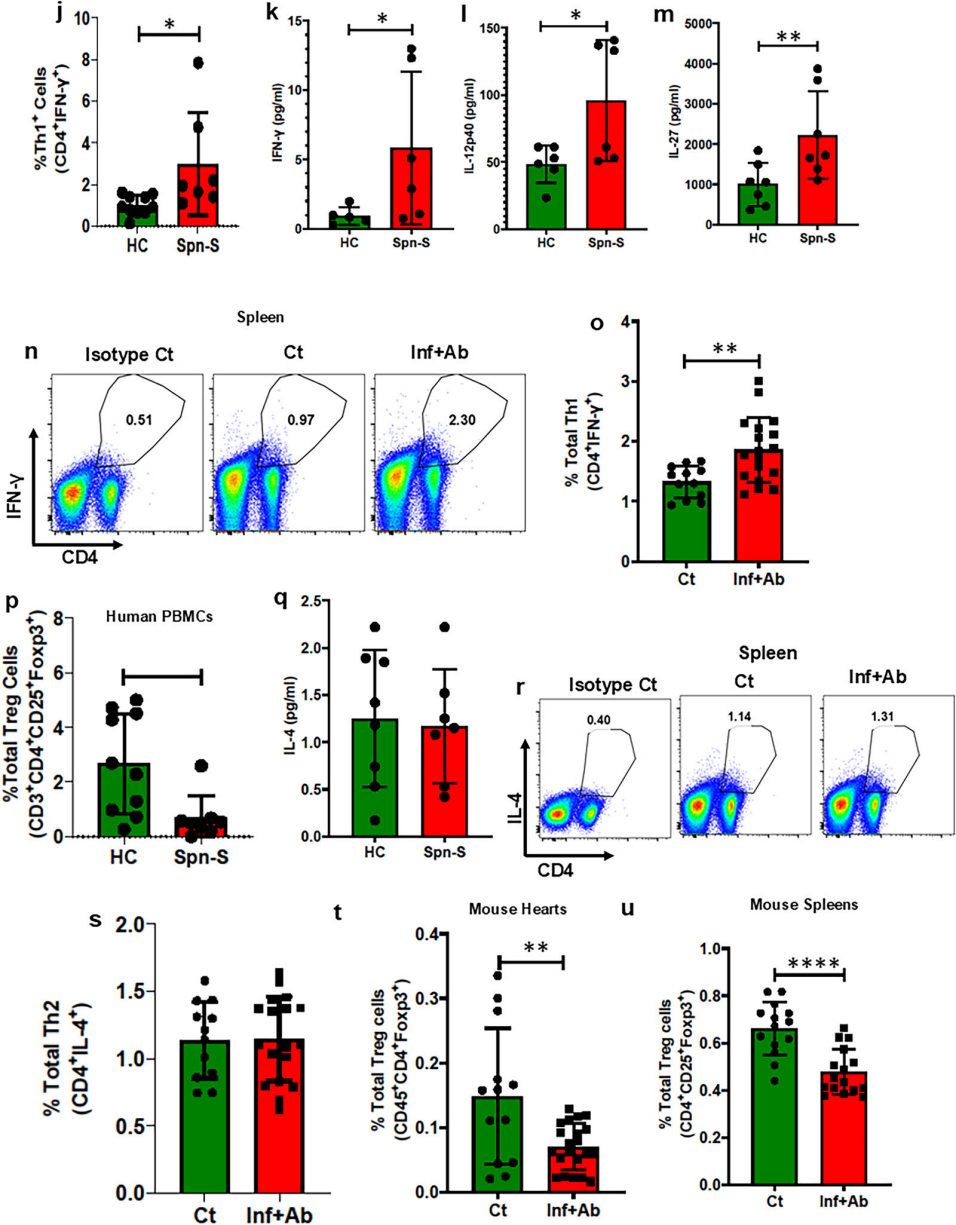
Consistent activation of T cells, induced pathogenic Th1 polarization and reduced T_reg_ frequency contribute to persistent inflammation in survivors of clinical and preclinical *Spn* infection. **a** Human serum IL-15 was measured by multiplex assay (*N* = 6–8). PBMCs were isolated from both healthy individuals and those who survived pneumonia. Subsequently, flow cytometry analysis was conducted to determine the percentage of various subpopulations. **b** A representative flow plot demonstrating CD69^+^CD4^+^ T cells. **c** The percentage of total CD4^+^CD69^+^ T cells (*N* = 7–10). **d** A representative flow plot demonstrating CD69^+^CD8^+^ T cells. **e** The percentage of total CD8^+^CD69^+^ T cells (*N* = 7–10). **f** A representative flow plot demonstrating CD25^+^CD4^+^ T cells. **g** The percentage of total CD4^+^CD25^+^ T cells (*N* = 7–10). **h** Human serum CXCL-9 was measured by multiplex assay (*N* = 7–8). **i** Human serum CXCL-10 was measured by multiplex assay (*N* = 7–8). **j** The percentage of total Th1 cells (*N* = 7–10). **k** Human serum IFN-γ measured by multiplex assay (*N* = 5–6). **l** Human serum IL-12p40 measured by multiplex assay (*N* = 6). **m** Human serum IL-27 measured by multiplex assay (*N* = 7). **n** Representative flow plot demonstrating Th1 cells in the spleen of mice. **o** The percentage of total Th1 cells in the spleen of mice (*N* = 12–18, *N* = 8). **p** The percentage of total T_reg_ cells measured from human PBMCs (*N* = 7–10). **q** Human serum IL-4 measured by multiplex assay (*N* = 7–8). **r** A representative flow plot demonstrating Th2 cells in the spleen of mice. **s** The percentage of total Th2 cells in the spleen of mice (*N* = 12–18). **t** The percentage of total T_reg_ cells measured with isolated leukocytes from the digested heart of mice (*N* = 13–18). **u** The percentage of total T_reg_ cells measured from the spleen of mice (*N* = 14–18). *P < 0.05, **P < 0.01, ***P < 0.001, ****P < 0.0001. The data are presented as mean ± standard deviation, and the significance of the data was determined using a two-tailed Mann–Whitney *U* test.

T cells are known to exert a pivotal role in inflammation-induced cardiac remodeling and dysfunction in chronic disease models such as pressure overload^35^. Suggestive of the involvement of T cell dynamics in the persistent inflammation following the *Spn* infection phase, we observed elevated levels of IL-15, which induces proliferation and activation of T cells in *Spn*-surviving humans (Fig. 6a). Indeed, evidence of T cell activation was observed following staining for CD69 and CD25 activation markers in CD4^+^ and CD8^+^CD3^+^ cells (Fig. 6b–g). Further investigation revealed increased levels of the Th1 helper T cells polarizing chemokines CXCL-9 and CXCL-10 as well as an increased frequency of proinflammatory Th1 cells in *Spn*-survived patients (Fig. 6h–j). Moreover, Th1 signature cytokines such as IFN-γ, along with the Th1 polarizing cytokines IL-12 and IL-27, were upregulated in *Spn*-survived patients (Fig. 6k–m). Quite strikingly, our analyses of mouse splenocytes generated data entirely consistent with the findings made using human samples (Fig. 6n,o and Supplementary Fig. 4). Notably, no differences in serum IL-4 levels, a signature cytokine of anti-inflammatory Th2 cells, were observed among *Spn*-survived patients. Moreover, we observed a reduced frequency of other anti-inflammatory helper T subsets, specifically T_reg_ cells, using PBMCs obtained from patient samples (Fig. 6p). Similar results were observed for Th2 and T_reg_ cells in the spleens of *Spn*-survived mice when comparable with their respective controls (Fig. 6q–u and Supplementary Figs. 4 and 5). Taken together, these data suggest that the activation of T cells, coupled with impaired dynamics of anti-inflammatory Th1/Th2/T_reg_ subsets, is likely to be a major contributor to T cell-mediated excessive inflammation in patients who have survived IPD.

Next, we sought to determine whether an *Spn* antigen-specific T cell response persists in IPD-survived animals even after bacterial clearance. To investigate this, we conducted ex vivo coculture experiments using immunosorted T cells and myeloid cells at a 10:1 ratio, stimulating them with *Spn*-derived complete soluble antigen. Cytotoxicity assays using annexin V and 7AAD revealed that antigen-presenting cells from IPD-survived animals exhibited reduced cell death compared with control groups (Supplementary Fig. 6a–c). In addition, BrdU incorporation in coculture experiments further demonstrated that antigen-specific myeloid cells and T cells, including CD4^+^ and CD8^+^ T cells, proliferated and became activate in response to *Spn* complete soluble antigen stimulation (Supplementary Fig. 7a–j). However, proliferation of antigen-specific T_reg_ cells was found to be impaired in IPD-survived animals (Supplementary Fig. 7k). Collectively, these findings indicate that an antigen-specific T cell response persists in IPD-survived animals even 3 weeks after bacterial clearance.

B cells play a crucial role in supporting and modulating T cell-mediated adaptive immune responses^36^. Consistent with patient data (Fig. 2h), we observed an increased frequency of splenic and cardiac B cells in IPD-survived mice compared with control groups (Supplementary Fig. 8a–c). In addition, the reduced levels of anti-inflammatory cytokines (IL-10 and TGF-β), along with diminished B cell-mediated IL-10 and TGF-β production, suggest that B cells also contribute to sustaining chronic inflammation (Supplementary Fig. 8d–h).

### Anti-inflammatory therapy with hydrocortisone rescued heart function in *Spn*-survived animals

Our findings with patient samples and in an animal model unequivocally demonstrate a persistent proinflammatory state in convalescent individuals and animals recovering from severe *Spn* infection. As chronic inflammation is known to be a critical factor for the development of cardiac remodeling and dysfunction, we hypothesized that reducing inflammation would prevent IPD-induced cardiac dysfunction. Such a result would support the notion that *Spn*-induced residual inflammation was at fault for postinfection MACE and provide a strong rationale for the testing of anti-inflammatory agents as potential prophylactic therapies to prevent MACE in convalescence.

**Fig. 7:**
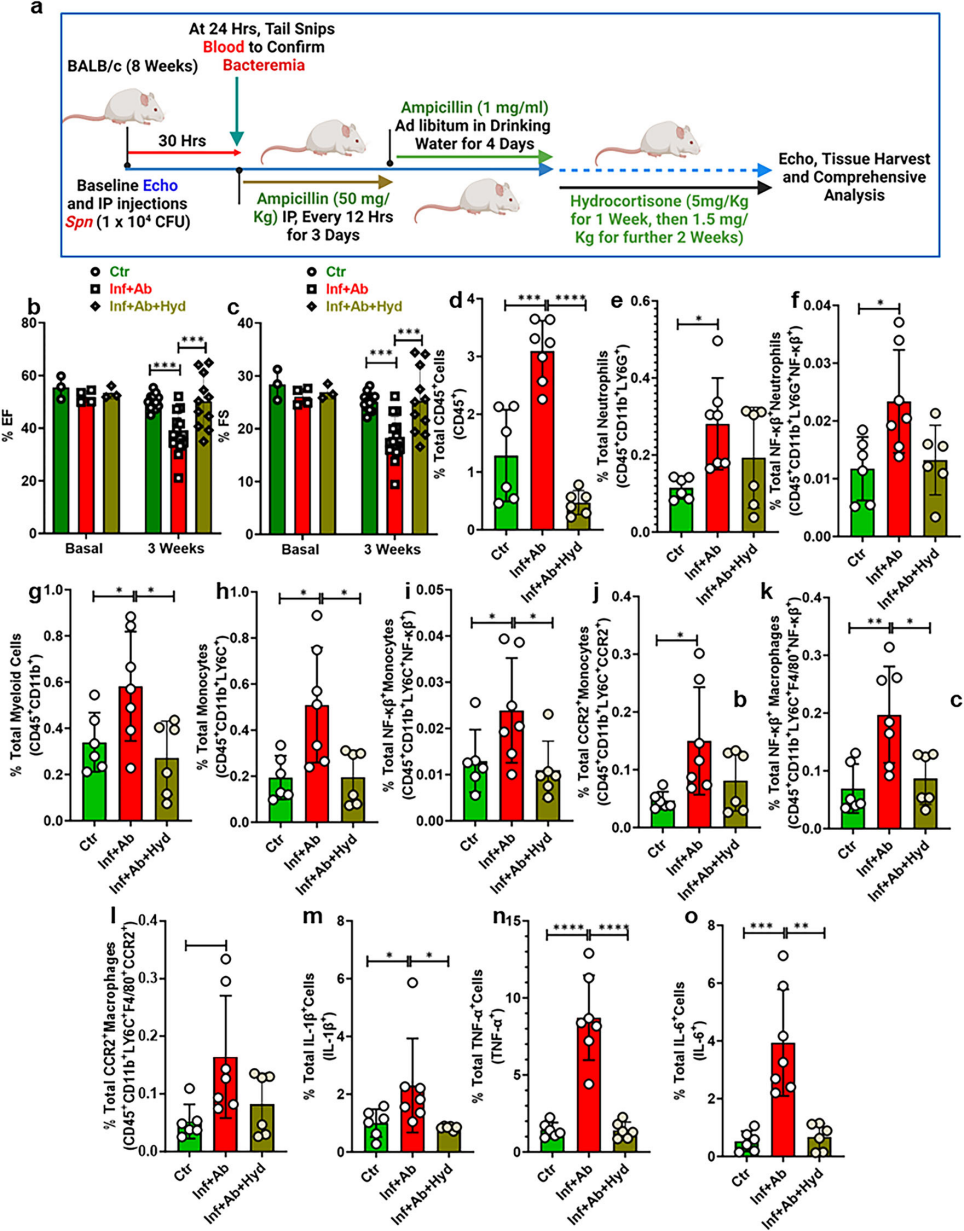
Hydrocortisone preserves heart function in surviving animals infected with *Spn* via immunosuppression. **a** A schematic diagram illustrating the approach to demonstrate the administration of hydrocortisone in mice. Ct, control (naive animals); Inf+Ab, infected then antibiotic-treated animals; Inf+Ab+Hyd, infected then antibiotic + hydrocortisone-treated animals. **b** Ejection fraction (EF) (*N* = 3–11). **c**, Fraction shortening (FS) (*N* = 3–11). The left ventricles (LV) of the hearts of control, infected and antibiotic-treated and infected and antibiotic + hydrocortisone-treated animals were digested to create a single-cell suspension. Subsequently, these cells were stained with a panel of antibodies, and flow cytometry was performed. **d**–**n** Total percentages of CD45^+^ cells (*N* = 6) (**d**), CD11b^+^LY6G^+^ neutrophils (*N* = 6) (**e**), pNF-ƙβ^+^ neutrophils (*N* = 6-7) (**f**), CD45^+^CD11b^+^ myeloid cells (*N* = 6) (**g**), CD45^+^CD11b^+^LY6C^+^ monocytes (*N* = 6–7) (**h**), pNF-ƙβ^+^ monocytes (*N* = 6-7) (**i**), CD45^+^CD11b^+^LY6C^+^CCR2^+^ monocytes (*N* = 6–7) (**j**), pNF-ƙβ^+^ macrophages (*N* = 6–7) (**k**), CD45^+^CD11b^+^LY6C^+^F4/80^+^CCR2^+^ macrophages (*N* = 6-7) (**l**), IL-1β-producing cells (*N* = 4–7) (**m**), TNF-α-producing cells (*N* = 6-7) (**n**), IL-6-producing cells (*N* = 5–6) (**o**). *P < 0.05, **P < 0.01, ***P < 0.001, ****P < 0.0001. The data are presented as mean ± standard deviation, and the significance of the data was determined using a one-way ANOVA.

Due to their rapid action and high efficacy, glucocorticoids are a mainstay in the current management of immune-related adverse events^18^. As depicted in Fig. 7a, beginning at the time of antimicrobial administration, we administered hydrocortisone (5 mg/kg intraperitoneal) to *Spn*-survived animals four times a week, followed by a tapering regimen of 1.5 mg/kg for an additional 2 weeks^20,37^. Mice administered hydrocortisone had diminished loss of ejection fraction and fractional shorting, key cardiac function parameters. (Fig. 7b,c). Hydrocortisone-treated mice had markedly reduced frequency of CD45^+^ cells, myeloid cells, neutrophils, CCR2^+^ proinflammatory monocytes and macrophages, along with diminished activation of NF-ƙβ in neutrophils, myeloid cells and macrophages in the heart (Fig. 7d–l and Supplementary Figs. 9–12). Hydrocortisone treatment inhibited IPD-induced proinflammatory cytokines systemically as well (Fig. 7m–o and Supplementary Fig. 13). Thus, we observed that the dampening of inflammation in a therapeutic fashion protected against cardiac dysfunction.

### Paquinimod alleviated adverse cardiac remodeling without compromising metabolism in animals that survived *Spn* infection

**Fig. 8:**
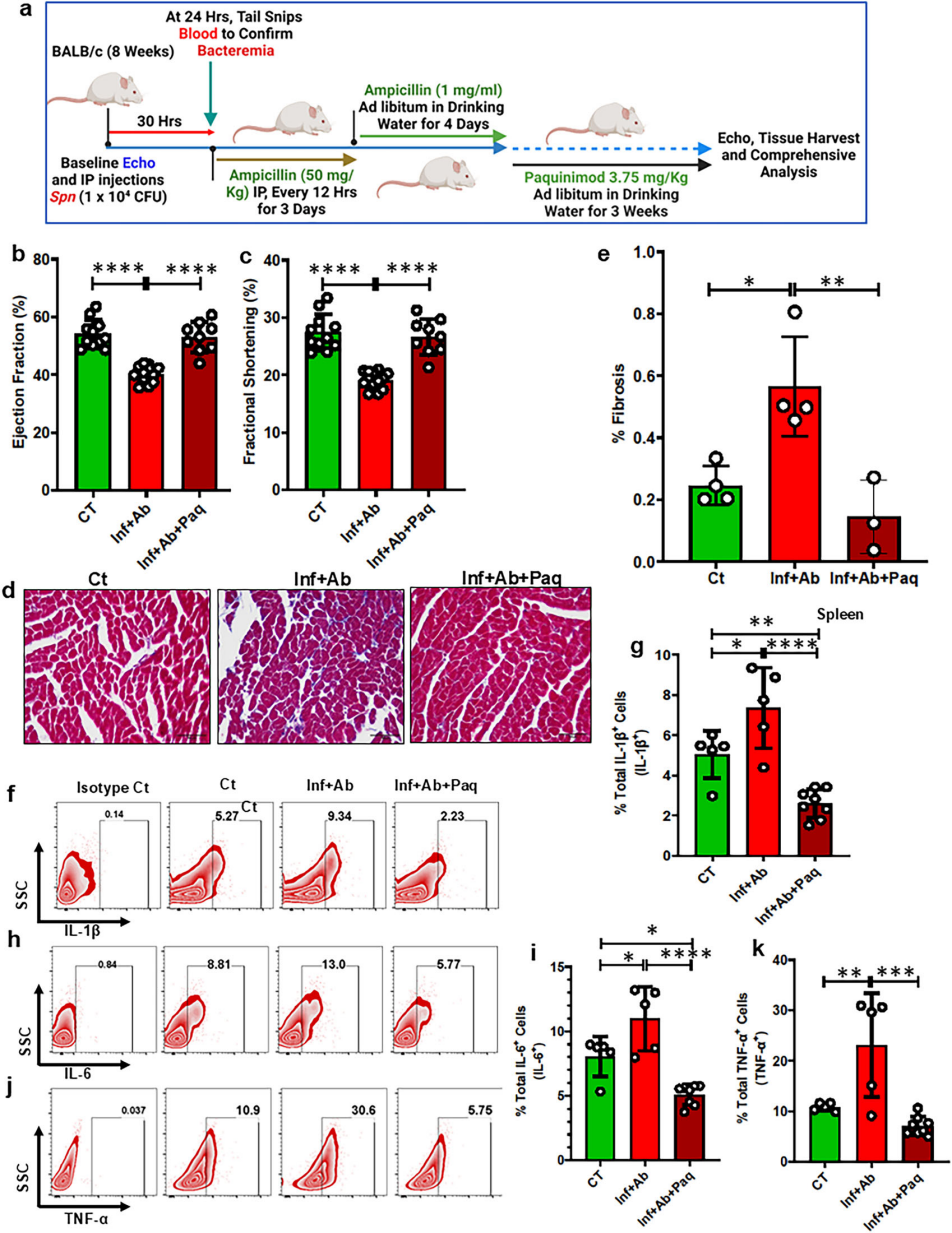

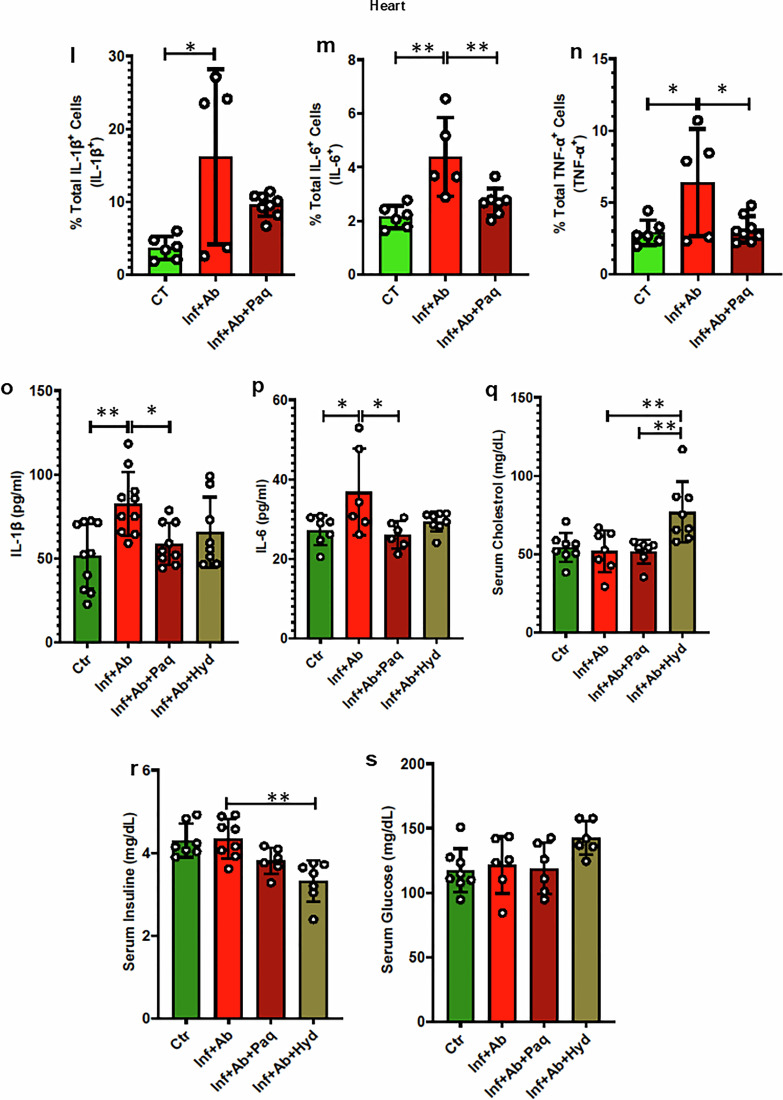
Paquinimod preserves heart function in surviving animals infected with *Spn* without impairing metabolism. **a** A schematic diagram illustrating the approach to demonstrate the administration of paquinimod in mice. Ct, control (naive animals); Inf+Ab, infected then antibiotic-treated animals; Inf+Ab+Paq, infected then antibiotic + paquinimod-treated animals. **b** Ejection fraction (EF) (*N* = 9–12). **c** Fraction shortening (FS) (*N* = 9–12). **d** Masson’s Trichrome staining in stained left ventricle (LV) regions (100 μm scale). **e** A quantification of LV fibrosis (*N* = 3–4). **f** A representative figure of flow cytometry showing the gating strategy to measure IL-1β^+^ cells from the spleen of all groups. **g** The data represent a quantitation of the percent of total IL-1β^+^ cells (*N* = 5–8). **h** A representative figure of flow cytometry showing the gating strategy to measure IL-6^+^ cells from the spleen of all groups. **i** The data represent a quantitation of the percent of total IL-6^+^ cells (*N* = 5–8). **j** A representative figure of flow cytometry showing the gating strategy to measure TNF-α^+^ cells from the spleen of all groups. **k** The data represent a quantitation of the percent of total TNF-α^+^ cells (*N* = 5–8). **l** The data represent a quantitation of the percent of total IL-1β^+^ cells (*N* = 5–8) in the heart. **m** The data represent a quantitation of the percent of total IL-6^+^ cells (*N* = 5–8) in the heart. **n** The data represent a quantitation of the percent of total TNF-α^+^ cells (*N* = 5–8) in the heart. **o** Serum IL-1β measured by ELISA (*N* = 7–10). **p** Serum IL-6 measured by ELISA (*N* = 6–8). **q** Serum cholesterol (*N* = 7–8). **r** Serum insulin (*N* = 7–8). **s** Serum glucose (*N* = 6–8). *P < 0.05, **P < 0.01, ***P < 0.001, ****P < 0.0001. The data are presented as mean ± standard deviation, and the significance was determined using a one-way ANOVA.

Our investigations indicated the excessive activation of the S100A8/9-NLRP3 signaling axis in *Spn*-treated animals. However, direct evidence that this signaling axis drives cardiac pathologies in *Spn*-rescued animals remained lacking. To determine this and ascertain if anti-inflammatory agents with greater specificity than glucocorticoids have promise, we designed in vivo rescue experiments with paquinimod, a specific inhibitor of alarmins (S100A9)^18^. The experimental design of the paquinimod studies is outlined in Fig. 8a. Indeed, intervention with paquinimod nearly abolished the detrimental cardiac effect of IPD (Fig. 8b–e). As anticipated, paquinimod treatment reduced the frequency of IL-1β-, IL-6- and TNF-α-producing cells in the spleen and myocardium (Fig. 8f–n and Supplementary Fig. 14). Notably, the body weight, HW/TL (Heart Weight to Tibial Length ratio) and cardiomyocyte surface area were all comparable in paquinimod treatment animals and controls (Supplementary Fig. 15).

We also compared the effects of paquinimod and hydrocortisone on regulating proinflammatory cytokines in *Spn*-survived animals. Both paquinimod and hydrocortisone treatments notably reduced IPD-induced plasma IL-1β and IL-6 (Fig. 8o, p). However, paquinimod did not affect M2 polarization and it substantially reduced the frequency of CD4^+^ T cells in *Spn*-survived mice (Supplementary Fig. 16a–d). Moreover, paquinimod treatment rescued the IPD-induced reduction in T_reg_, while exhibiting no effect on T cell proliferation (Supplementary Figs. 16e–i and 17). We also examined the potential metabolic consequences of proposed interventions. IPD itself did not induce any changes in plasma cholesterol, insulin and glucose levels, indicating that IPD exerts its adverse cardiac effects independently of these metabolic changes. However, treatment of the mice with hydrocortisone resulted in elevated plasma cholesterol and reduced insulin levels compared to the respective control groups (Fig. 8q–s). By contrast, intervention with paquinimod did not lead to any adverse metabolic effects (Fig. 8q–s). Thus, paquinimod treatment successfully rescued IPD-induced adverse cardiac effects without unwanted metabolic complications. Therefore, targeting this signaling axis could represent a novel therapeutic approach to prevent IPD-induced adverse cardiac events.

## Discussion

Lower respiratory tract infections are common and individuals who experience severe forms of pneumonia are at greater risk for MACE and cardiovascular events^1,2^. MACE during pneumonia contributes substantially to in-hospital mortality but also occurs at higher rates during convalescence and impacts mortality. Although the former can be attributed to acute physiological stress imposed by the infection, a molecular explanation for why individuals remain at risk in convalescence was lacking. The presented results are the first to provide a mechanistic explanation for this, tying unresolved persistent inflammation to increased CVD risk of patients with IPD in convalescence. Persistent unresolved inflammation, even after several weeks of antibiotic clearance of bacterial infection, was evident by multiple readouts, including elevated DAMPs, cytokines and chemokines in sera; differentiation of monocytes to proinflammatory monocytes and macrophages; increased production of monocytes and macrophages in BM and spleen; and T cell polarization into proinflammatory T cells. These findings were consistent in both humans recovering from pneumonia and our preclinical mouse model of *Spn* convalescence. Future studies with patients with *SPn* recruitment for follow-up studies to determine the correlation between ongoing chronic inflammation and cardiac dysfunction will be of meaningful interest.

Mechanistically, we identified S100A8/A9/TLR4/NLRP3 as a major contributor to unresolved inflammation, remodeling and cardiac dysfunction. This signaling axis is well-known to promote chemotaxis, immune cell proliferation and excessive inflammation. For the proof of concept, herein we employed immunomodulatory agents that dampened inflammation overall, that is, hydrocortisone, and those that specifically targeted activation of the S100A8/A9/TLR4/NLRP3 pathway, that is, paquinimod. Briefly, paquinimod works by blocking TLR4 recognition of DAMPS. Notably, the Gram-positive bacteria used in this study, *Spn*, are not recognized by TLR4; lipoteichoic acids in its cell wall are instead sensed by TLR1 and TLR2. Thus, our drug was not blocking TLR4 recognition of the bacterial products and most likely instead blocking recognition of DAMPS. As TLR4 is present on a wide variety of cell types, future studies with approaches to precisely identify the cell types (for example, neutrophils, macrophages or pathogenic T cells) driving cardiac remodeling are warranted to establish the cause-and-effect relationship of specific immune cell population recruitment in the heart tissue.

A detrimental role for unresolved inflammation following pneumonia is strongly supported by a recent phase 3 trial with 800 patients investigating the clinical use of hydrocortisone for patients with CAP^20^. In this multicentric, double-blind, randomized, controlled trial, patients with CAP received intravenous hydrocortisone (200 mg daily for either 4 or 7 days, followed by tapering for a total of 8 or 14 days) or placebo. All the patients received standard therapy, including antibiotics and supportive care. Encouragingly, patients who received hydrocortisone had a lower long-term risk of death than those who received a placebo. Echocardiography or cardiac damage markers were not measured in these patients; therefore, it is not possible to establish a direct association of the reported mortality pattern with the degree of cardiac damage. Nonetheless, this outcome, along with our results of preserved heart function in mice receiving hydrocortisone, suggests that the same may be occurring in humans. It will be critical to expanding on the results of our preclinical model showing that hydrocortisone and paquinomod suppressed excessive chronic inflammation and rescued cardiac dysfunction, doing so in human subjects.

Glucocorticoid treatment is not without risk, and hydrocortisone-treated patients with CAP demonstrated an increased incidence of hyperglycemia^38,39^. Consistent with this result, we also observed increased cholesterol, serum glucose and decreased serum insulin in hydrocortisone-treated animals. Thus, even though our interventional hydrocortisone studies are highly encouraging, alternative pharmacological agents with more precise action should instead be considered for clinical application. Remarkably, our results with paquinimod revealed that it could successfully mitigate the *Spn*-induced adverse cardiac effects without unwanted metabolic complications seen with hydrocortisone. Notably, paquinimod was found to be well tolerated in humans with only mild to moderate adverse effects^40,41^.

Notably, S100A8/9 was found to be required for the survival of pneumococcal pneumoniae in mice^42^. Similarly, S100A8A9 inhibition with paquinimod enhanced mouse morbidity and mortality in a *Pseudomonas aeruginosa* infection-induced cardiac dysfunction model^43^. In the latter, S100A8A/9-knockout animals consistently exhibited enhanced bacterial dissemination to the heart and worsened cardiac dysfunction^43^. These results indicate that the release of S100A8A/9 during infection is immunologically critical for controlling bacterial growth and recruiting the necessary immune defense against infection. Notably, we introduced paquinimod alongside antimicrobial therapy such that blocked TLR4 recognition of DAMPs occurred during and after pharmacological eradication of the bacteria—not during infection. We believe the vital differences in the treatment timeline are primarily responsible for the entirely contrasting outcomes in both studies. Therefore, the timeline of the suggested immunomodulatory therapies should be carefully determined to achieve the optimal clinical benefit.

In conclusion, our interventional studies with immunosuppressive agents hydrocortisone or pharmacological S100A9 inhibitor established the causative role of unresolved systemic and cardiac localized inflammation as being responsible for adverse cardiac remodeling and dysfunction. Along such lines, we have identified the central role of S100A8/A9-TLR-NLRP3 signaling mediated chronic unresolved inflammation in driving *Spn*-mediated cardiac pathologies in convalescence. Treatments for those hospitalized for *Spn* disease could be further optimized and tested for managing *Spn*-mediated cardiac pathologies in convalescence.

## Supplementary information

Supplementary Information
