# Supplementary material for: S100A8/9-NLRP3-mediated chronic unresolved inflammation drives cardiac pathologies following invasive pneumococcal disease
Source: Exp Mol Med. 2025 Oct 10;57(10):2344–63. doi: 10.1038/s12276-025-01552-8 (PMC12586492; doi:10.1038/s12276-025-01552-8)
Supplement: Supplementary file 1 — Supplementary Information [file 12276_2025_1552_MOESM1_ESM.pdf]

# Supplementary Figure 1

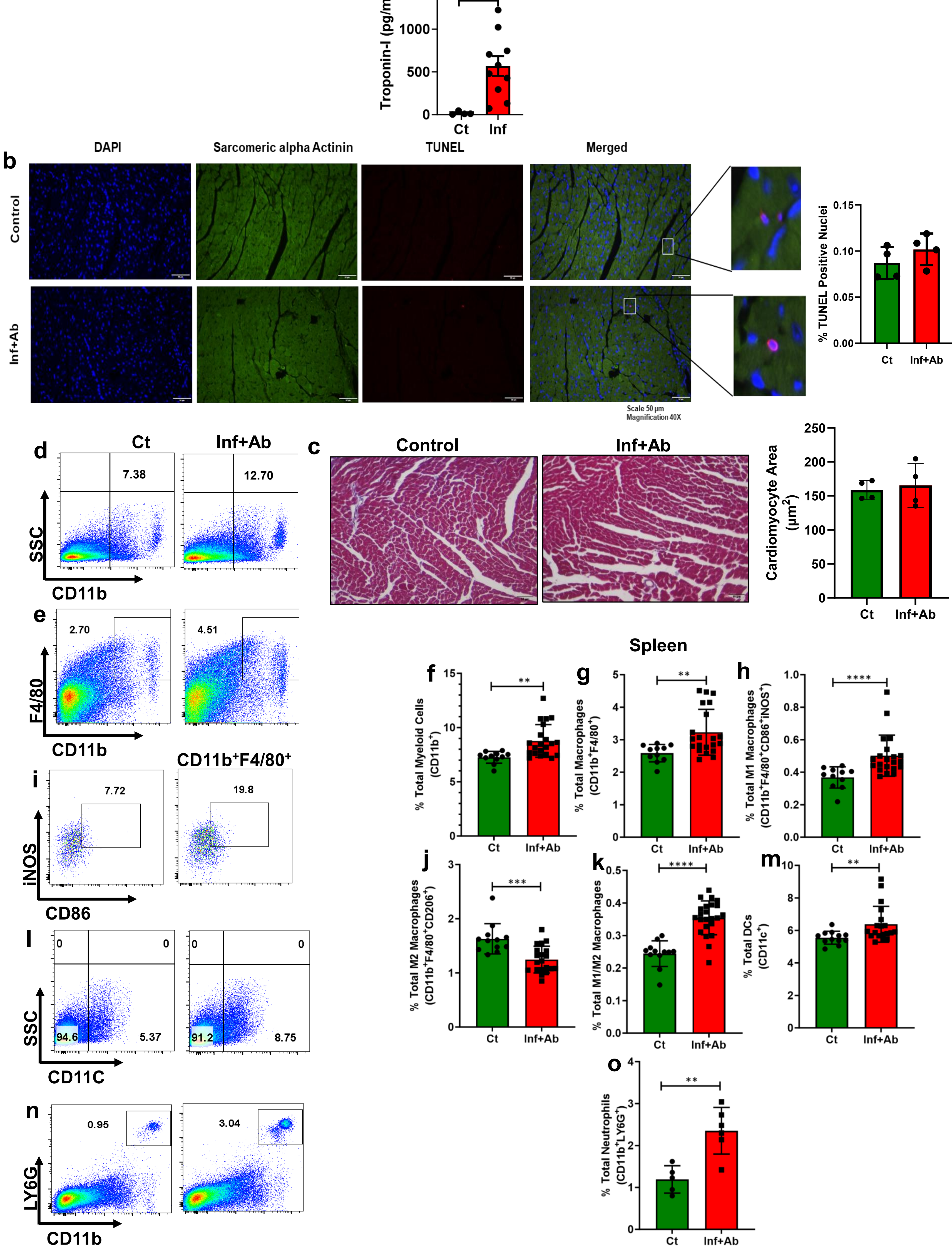

## Supplementary Figure 2

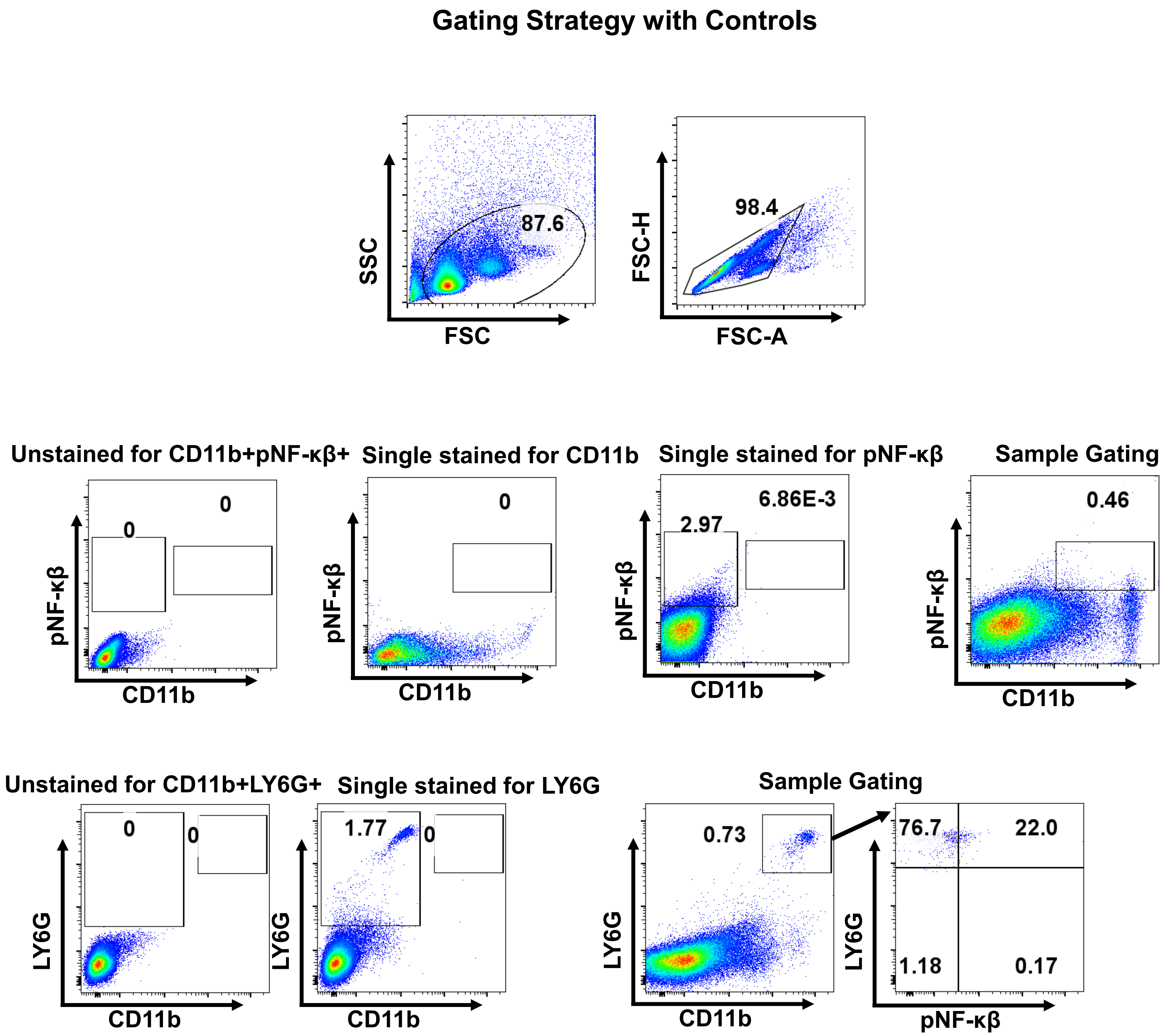

## Supplementary Figure 3

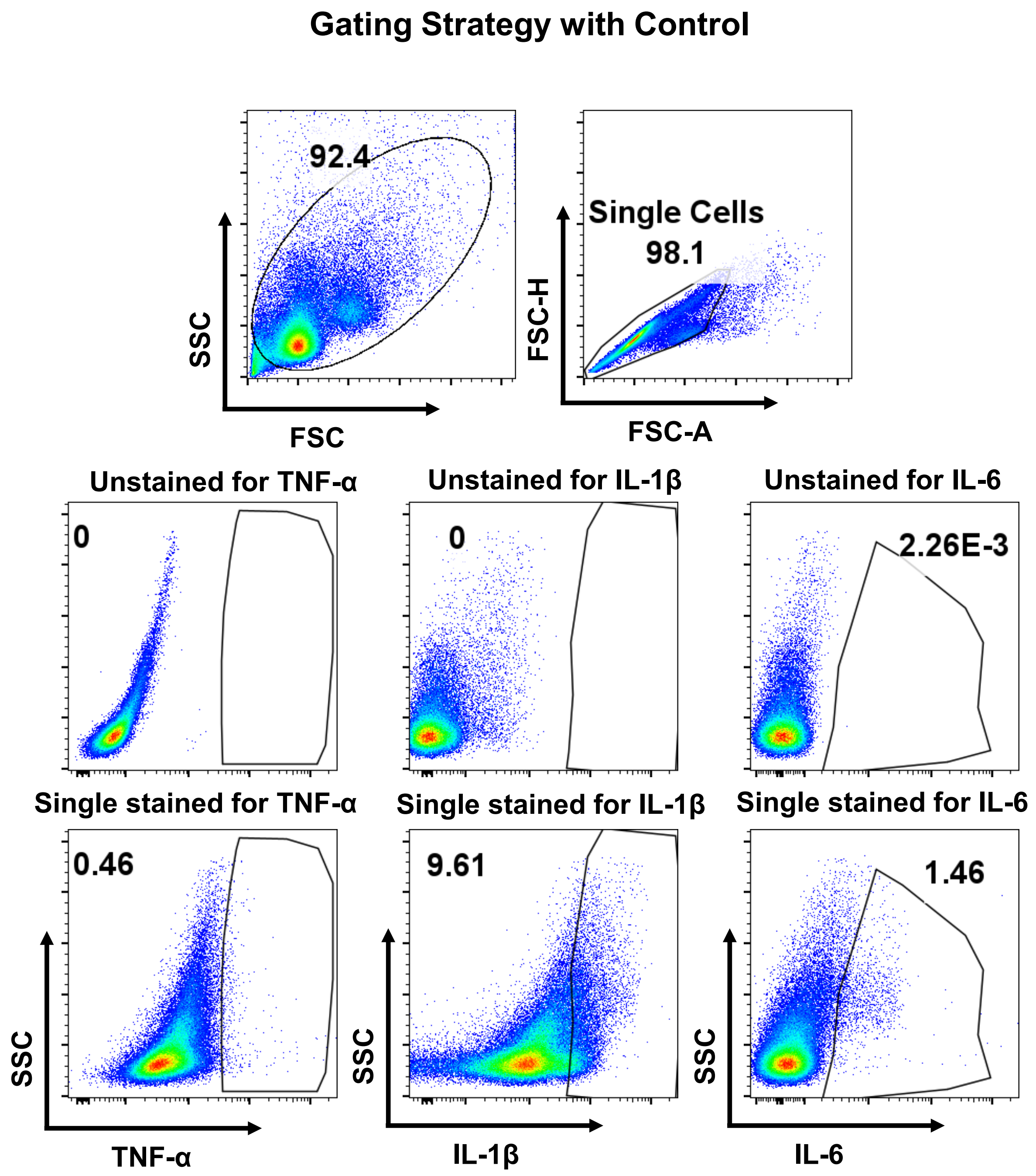

# Supplementary Figure 4

## Gating Strategy for Th1 and Th2 with Controls

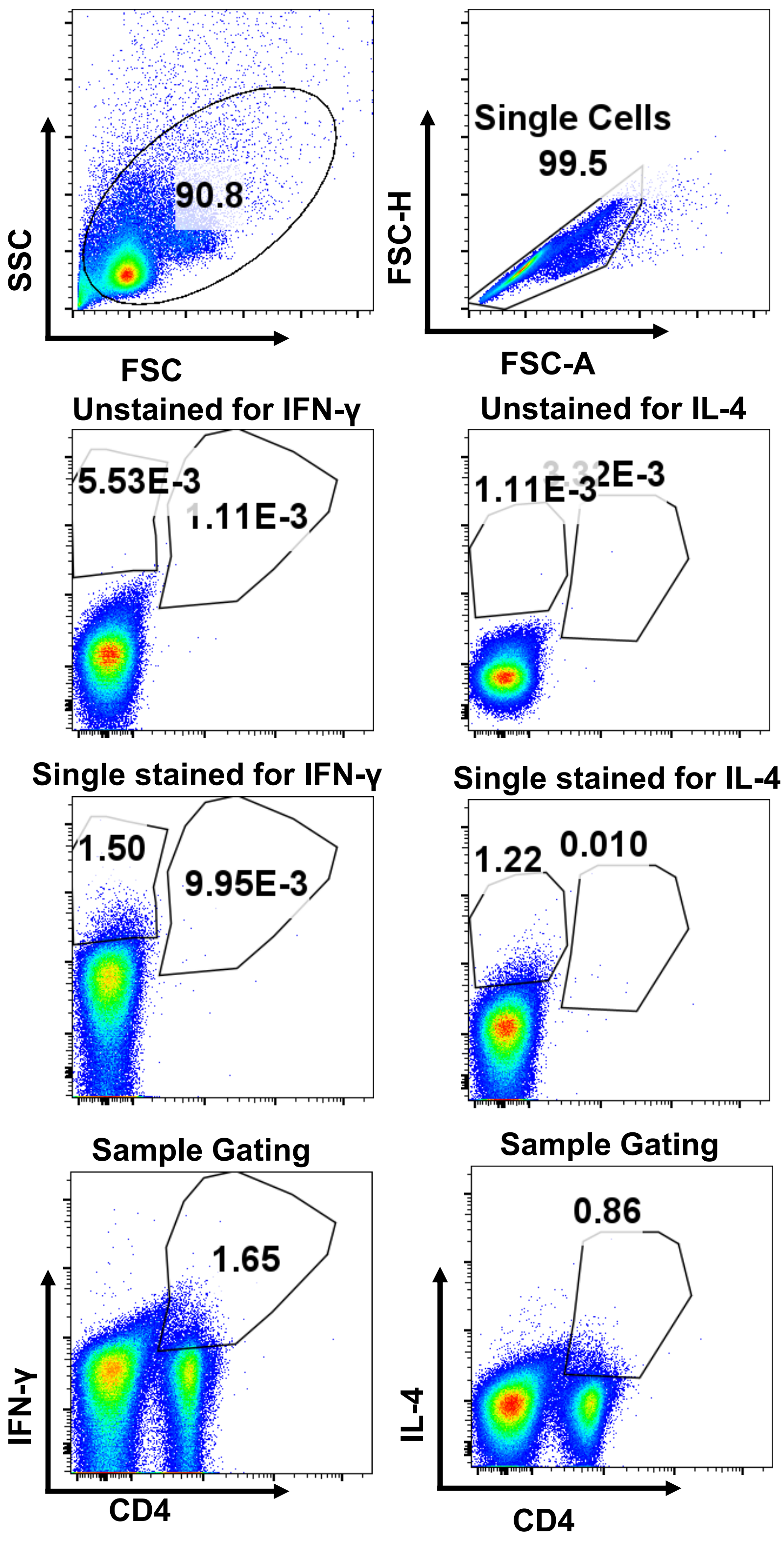

## Supplementary Figure 5

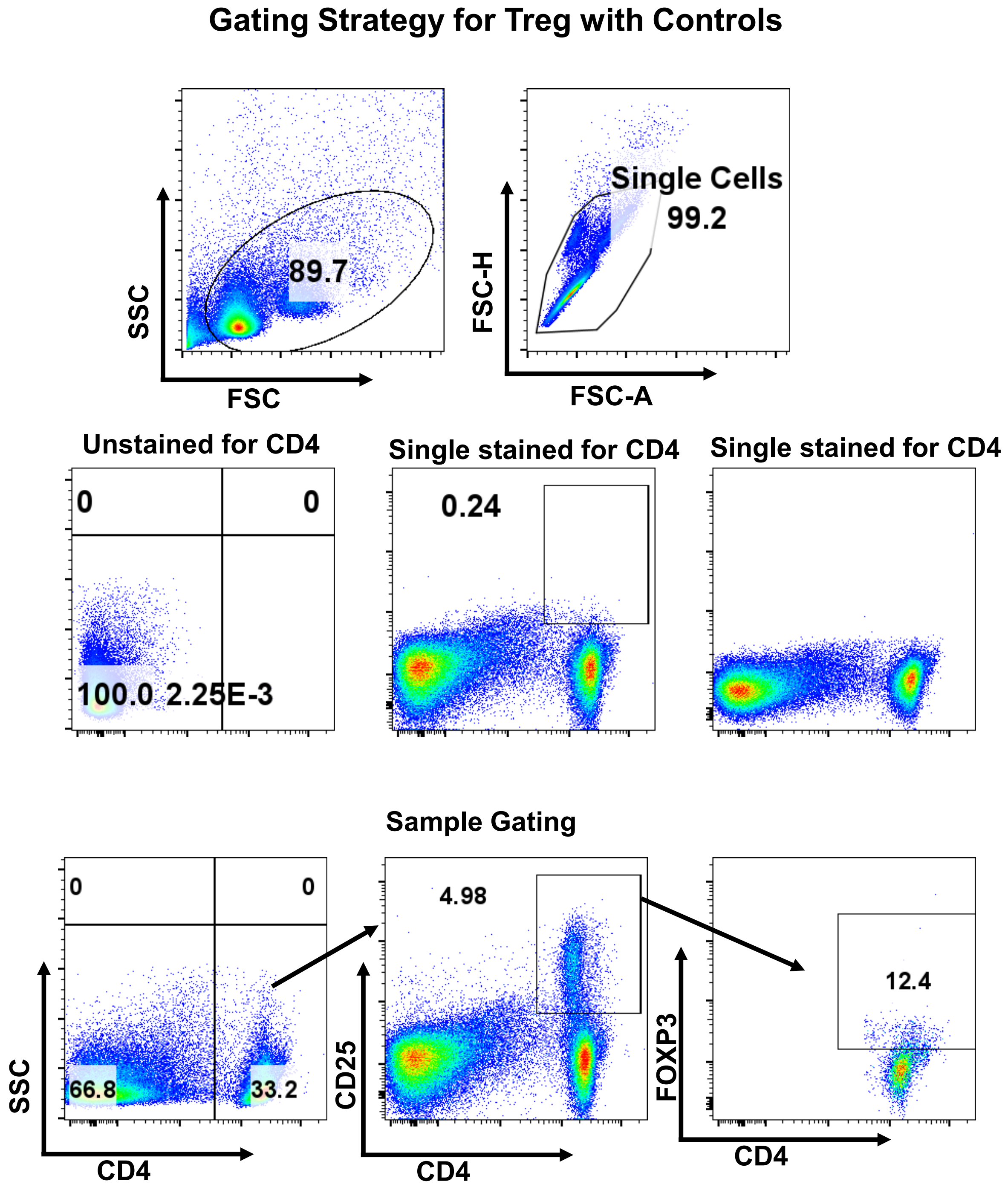

## Supplementary Figure 6

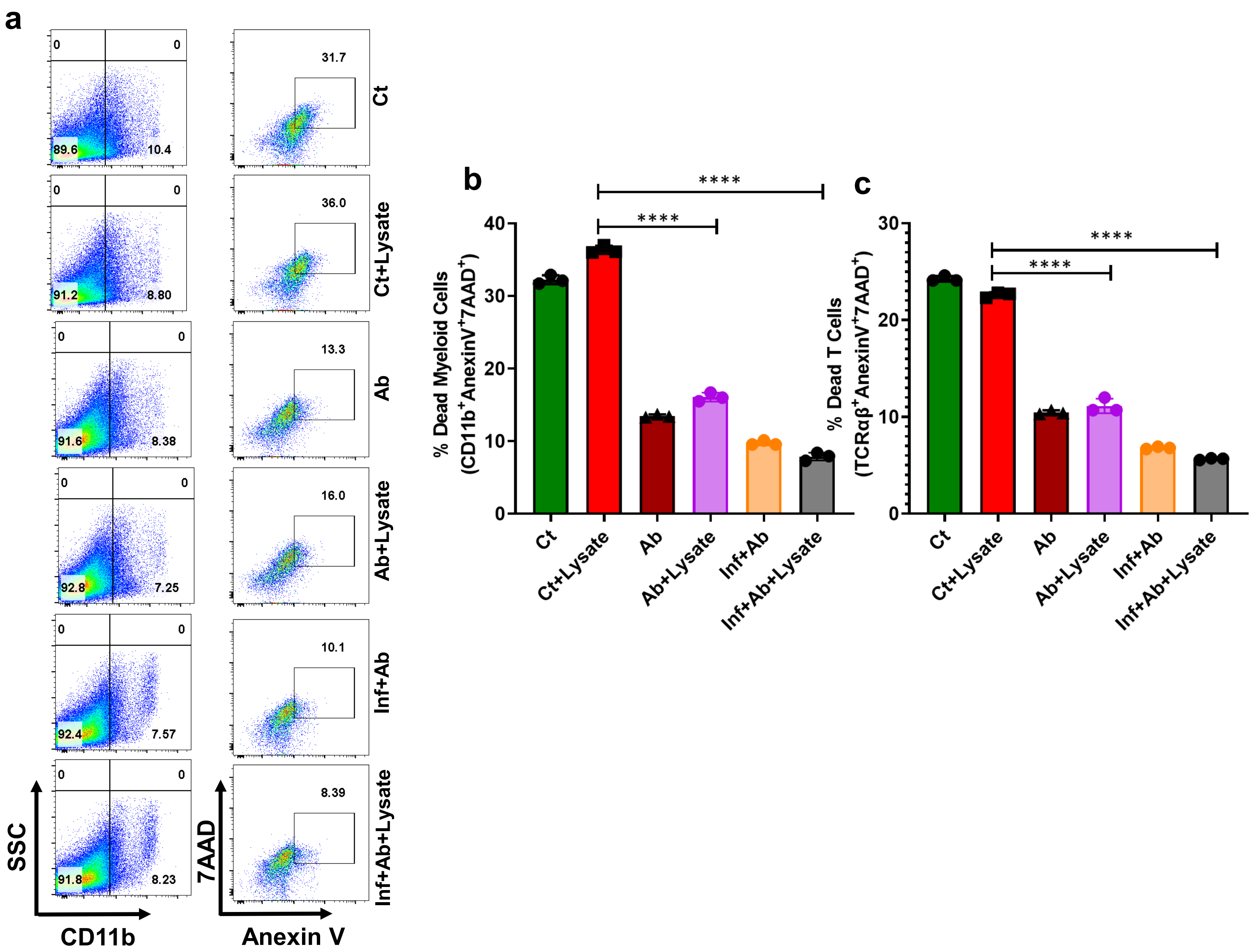

# Supplementary Figure 7

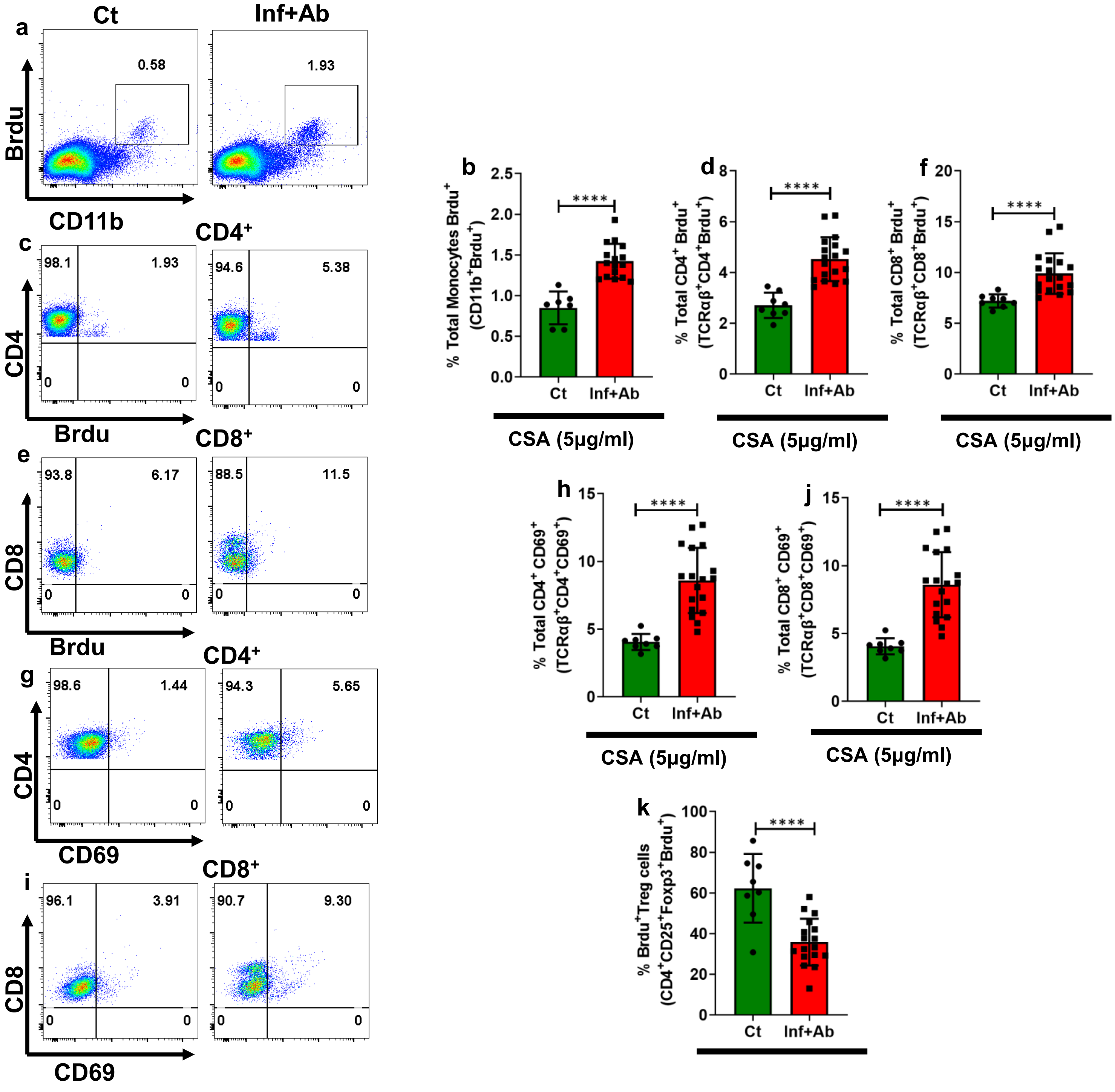

## Supplementary Figure 8

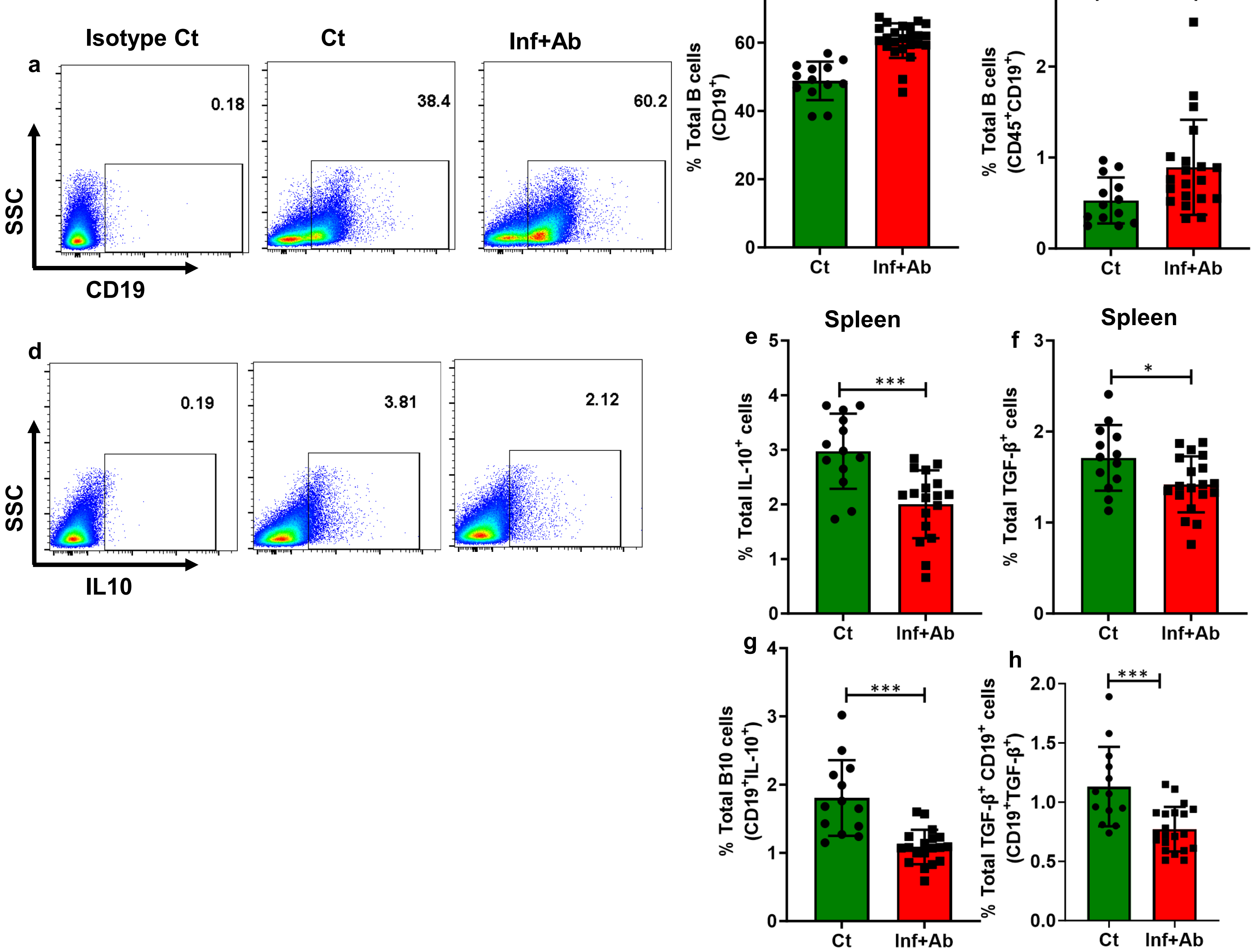

## Supplementary Figure 9

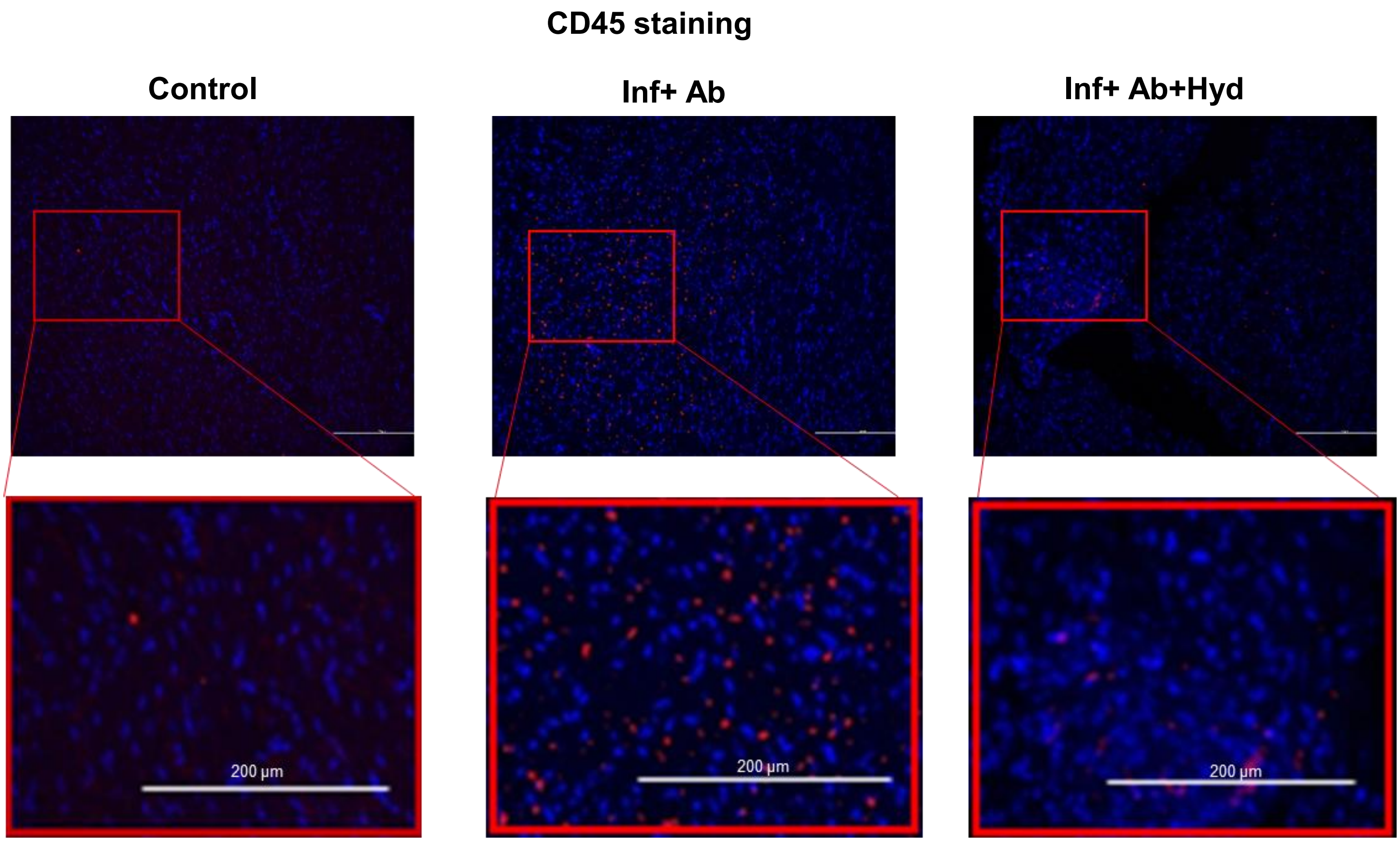

## Supplementary Figure 10

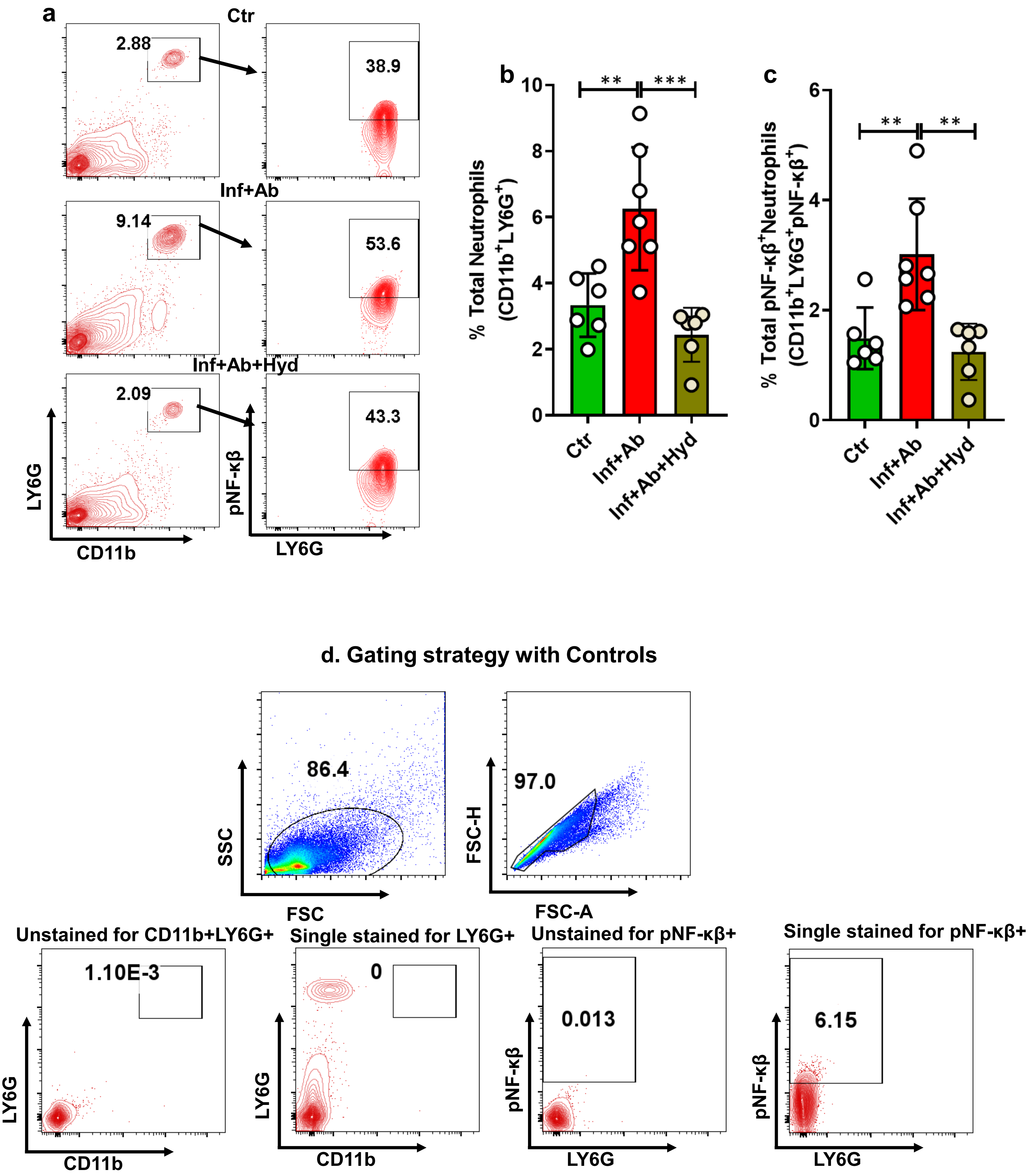

**Supplementary Figure 11**

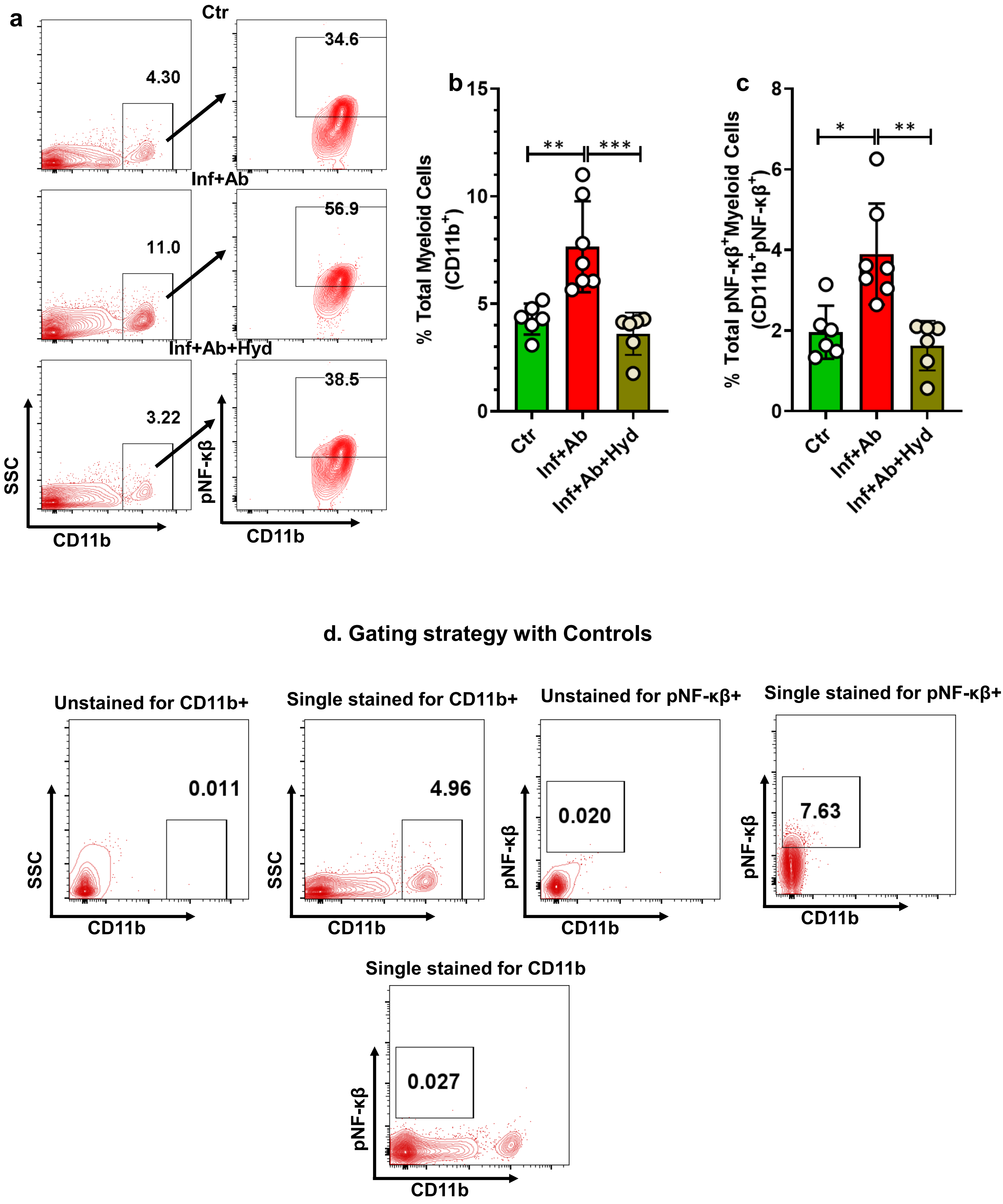

## Supplementary Figure 12

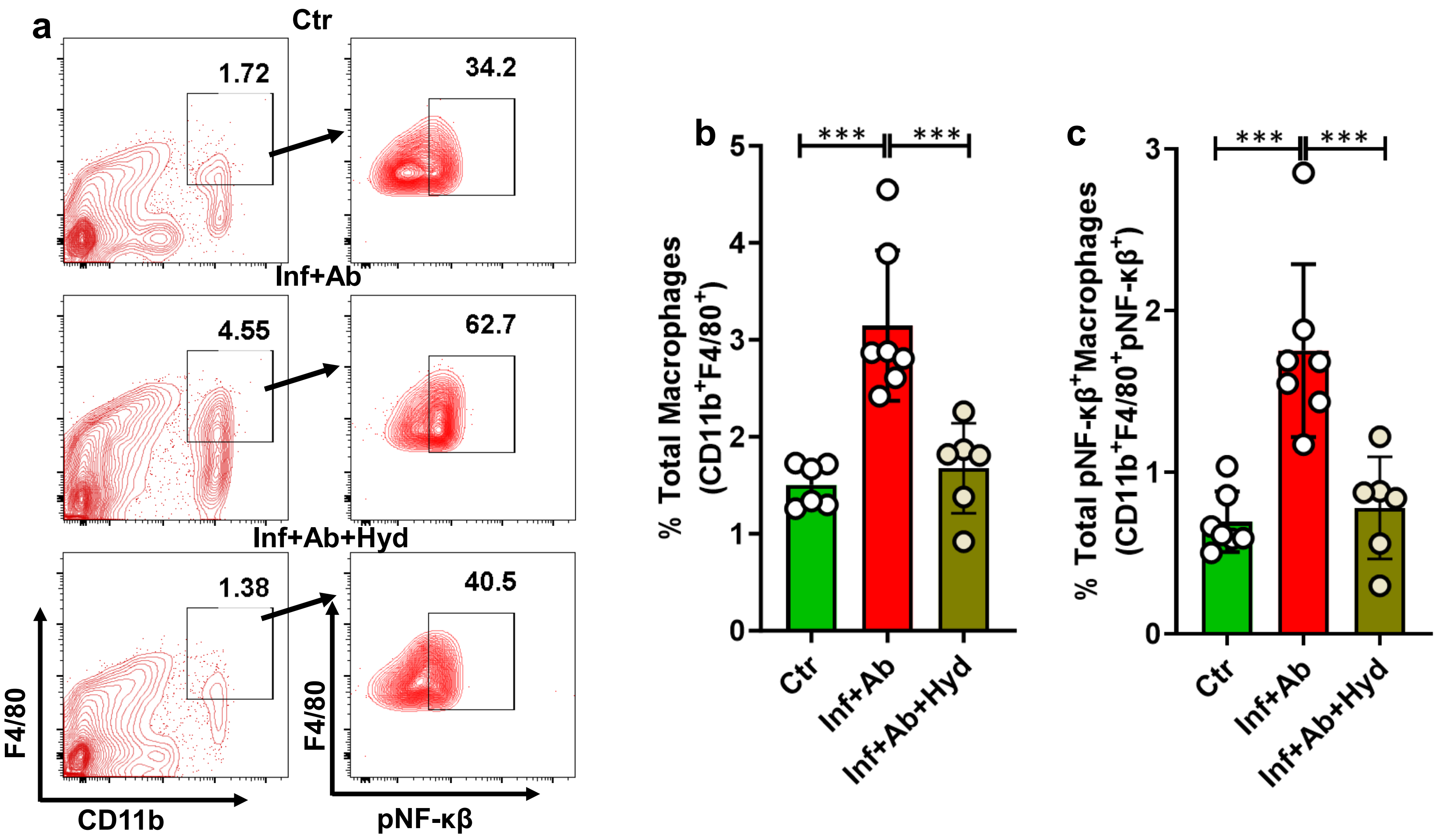


d. Gating strategy with Controls

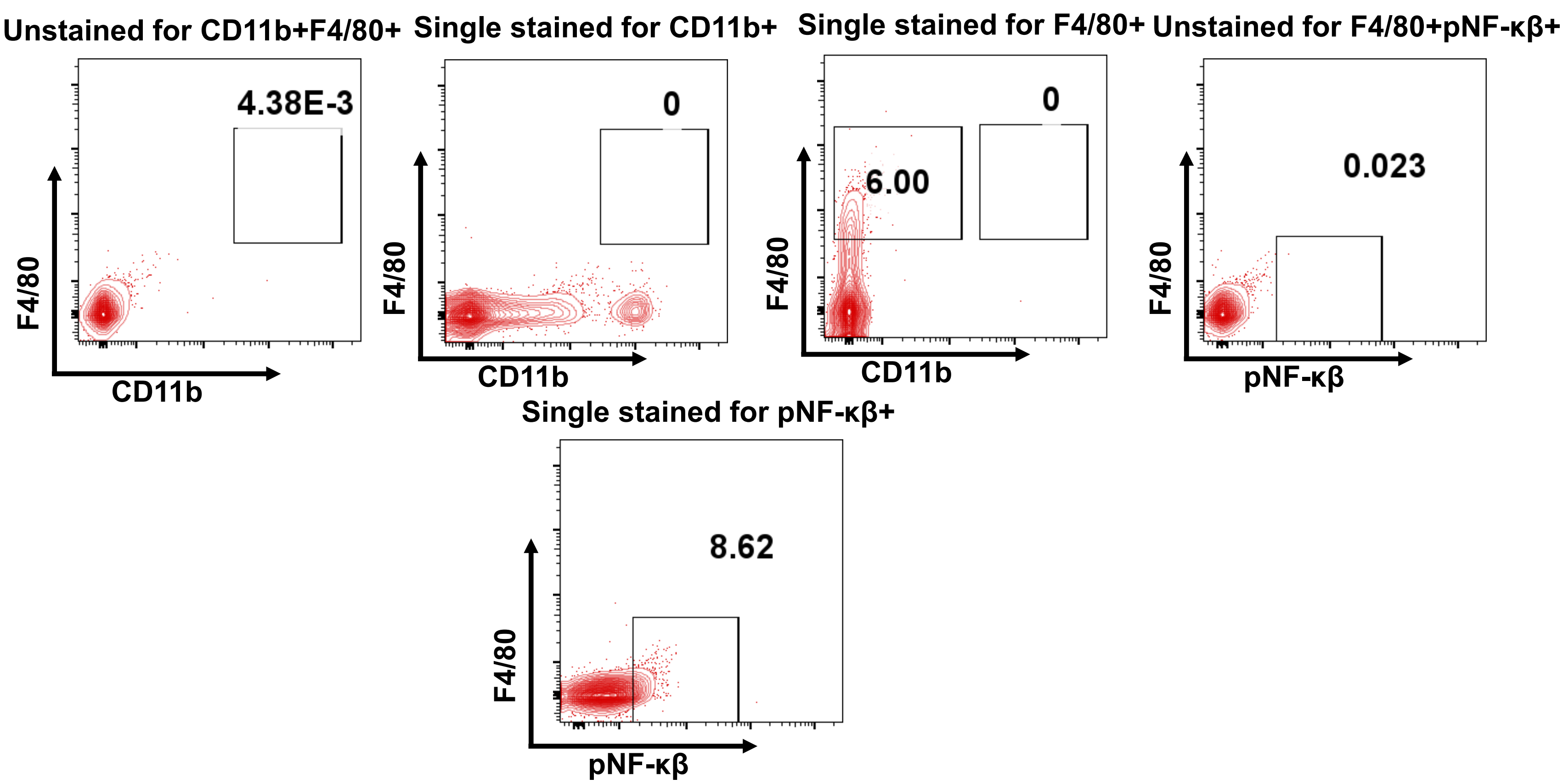

# Supplementary Figure 13

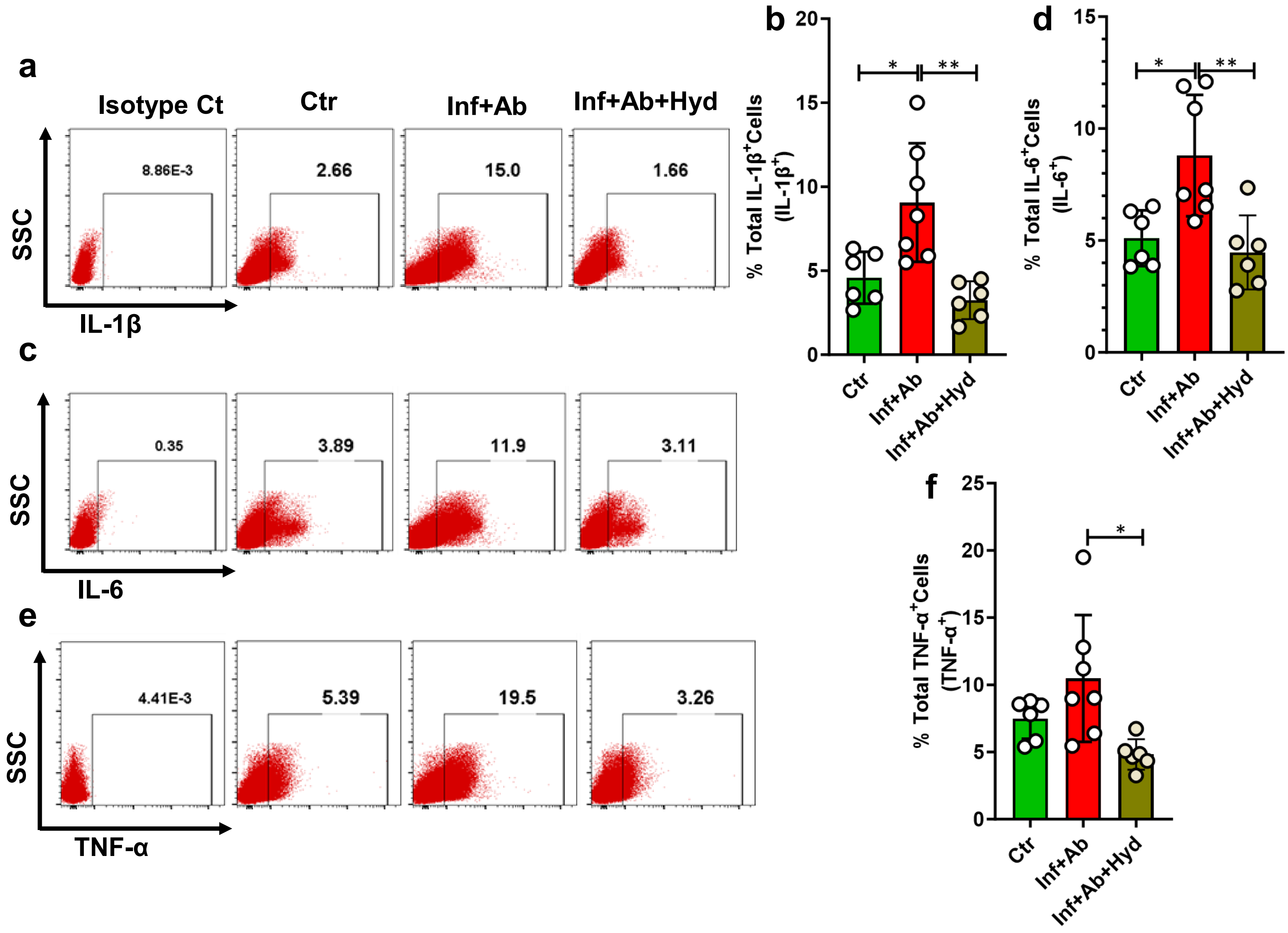

## Supplementary Figure14

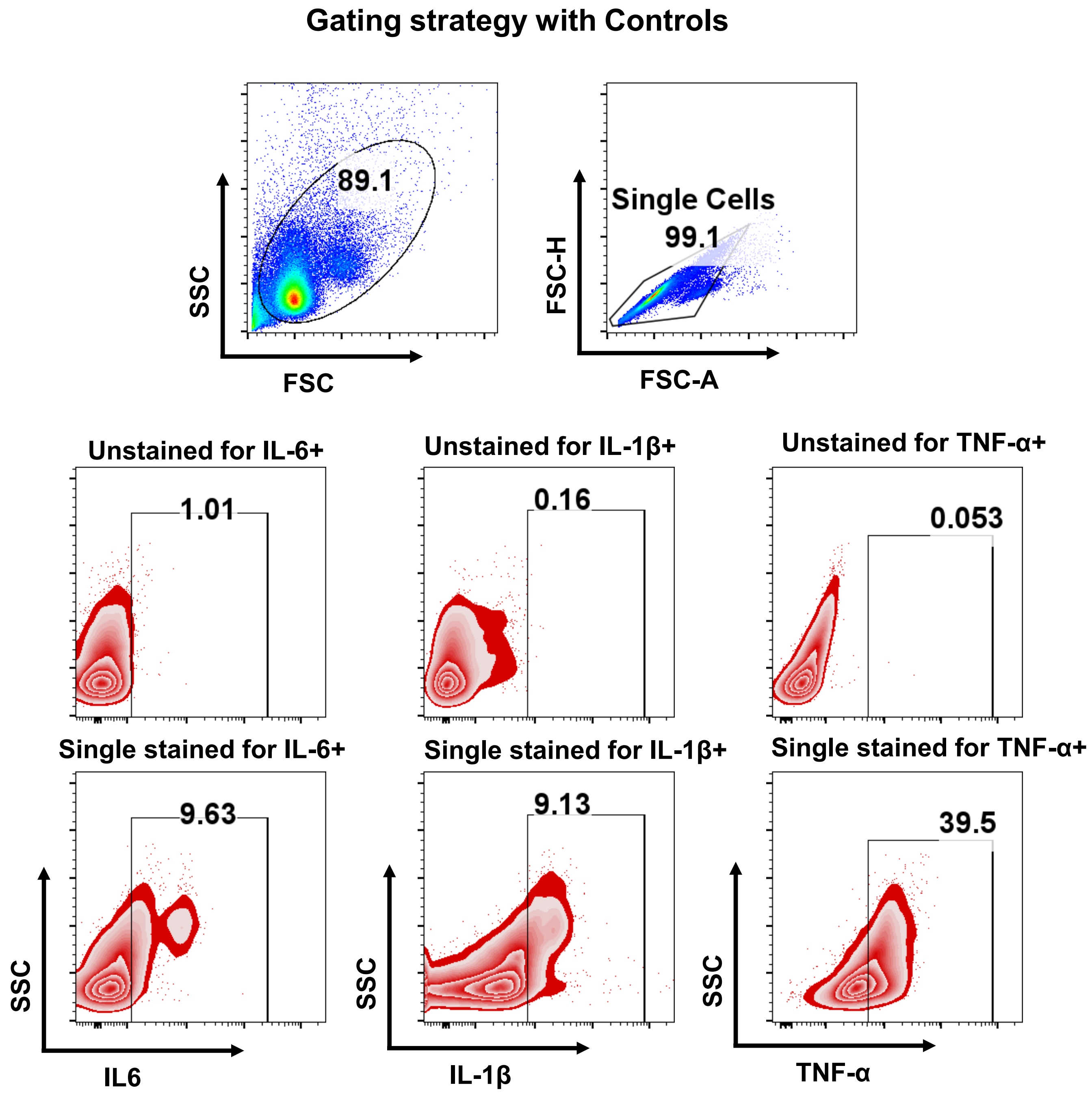

## Supplementary Figure 15

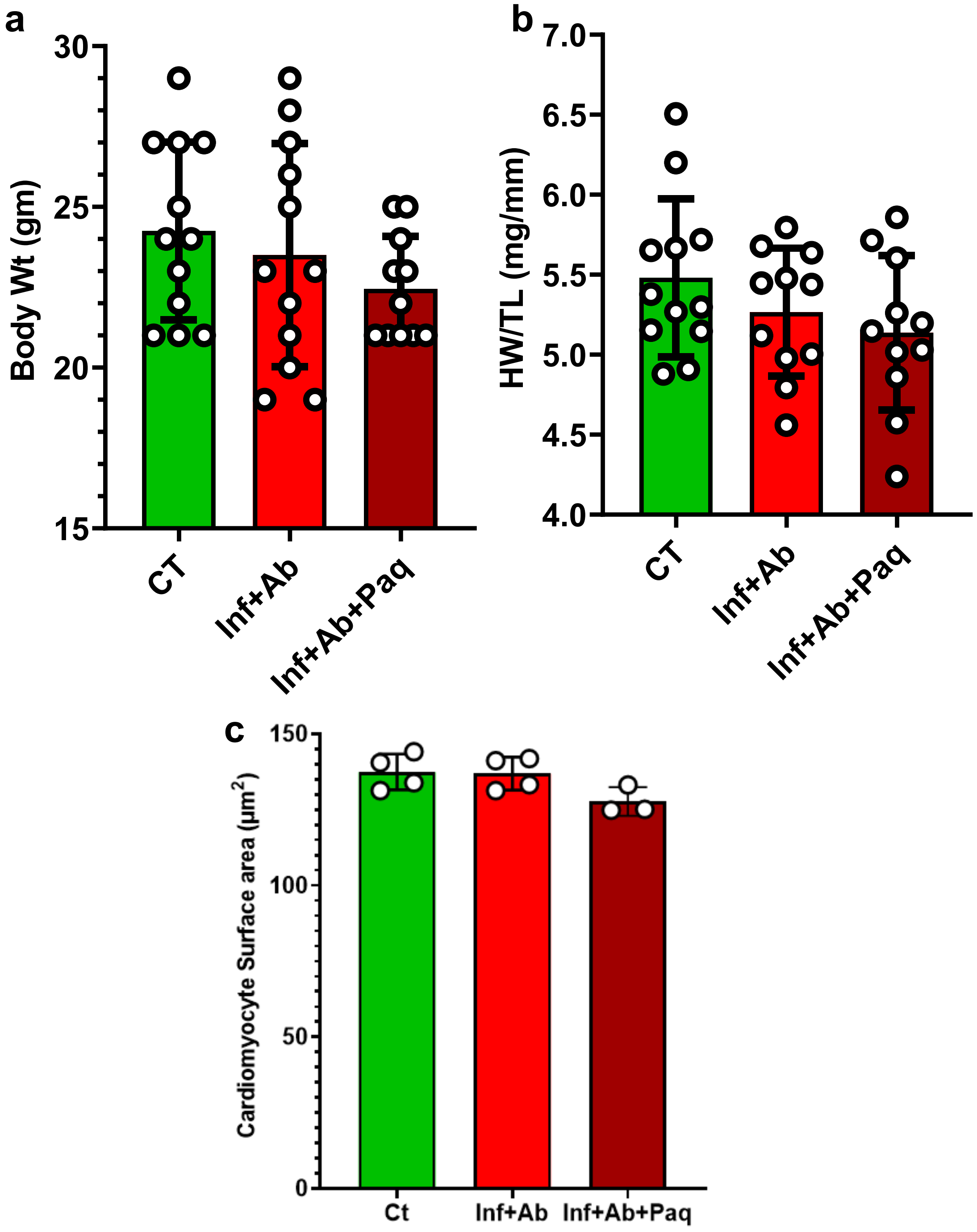

# Supplementary Figure 16

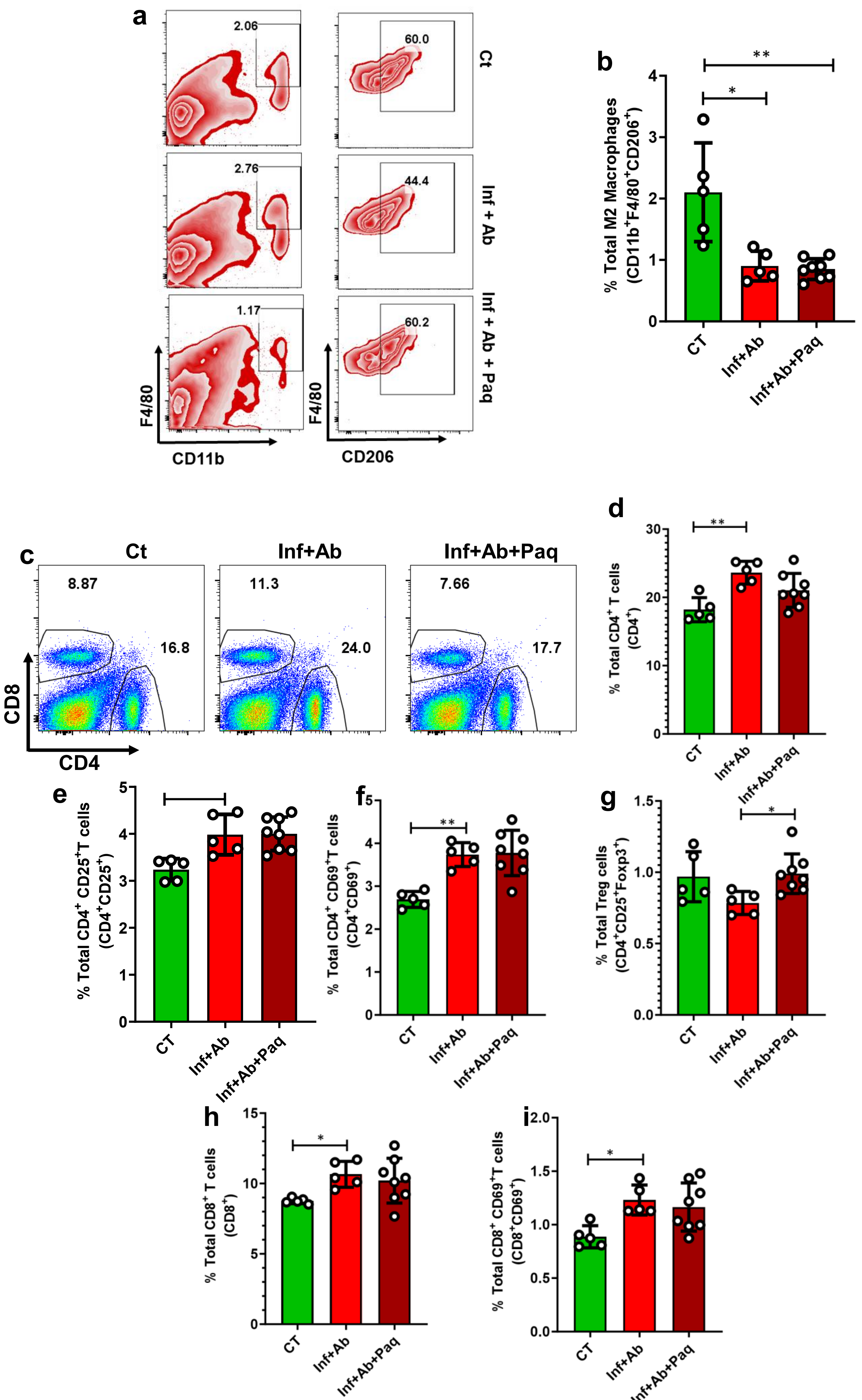

# Supplementary Figure 17

## a. Gating strategy for M2 with Controls

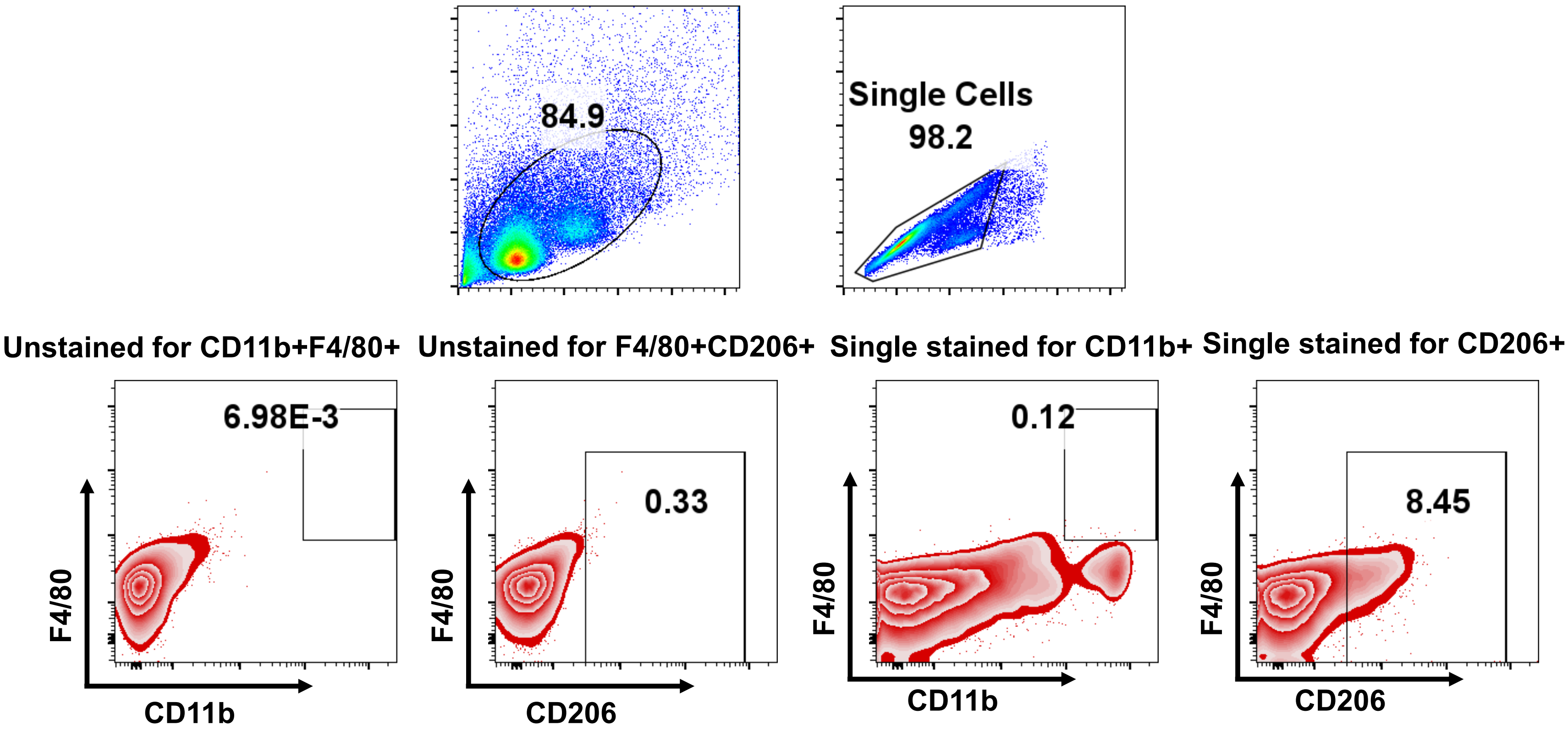


## b. Gating strategy for T cells with Controls

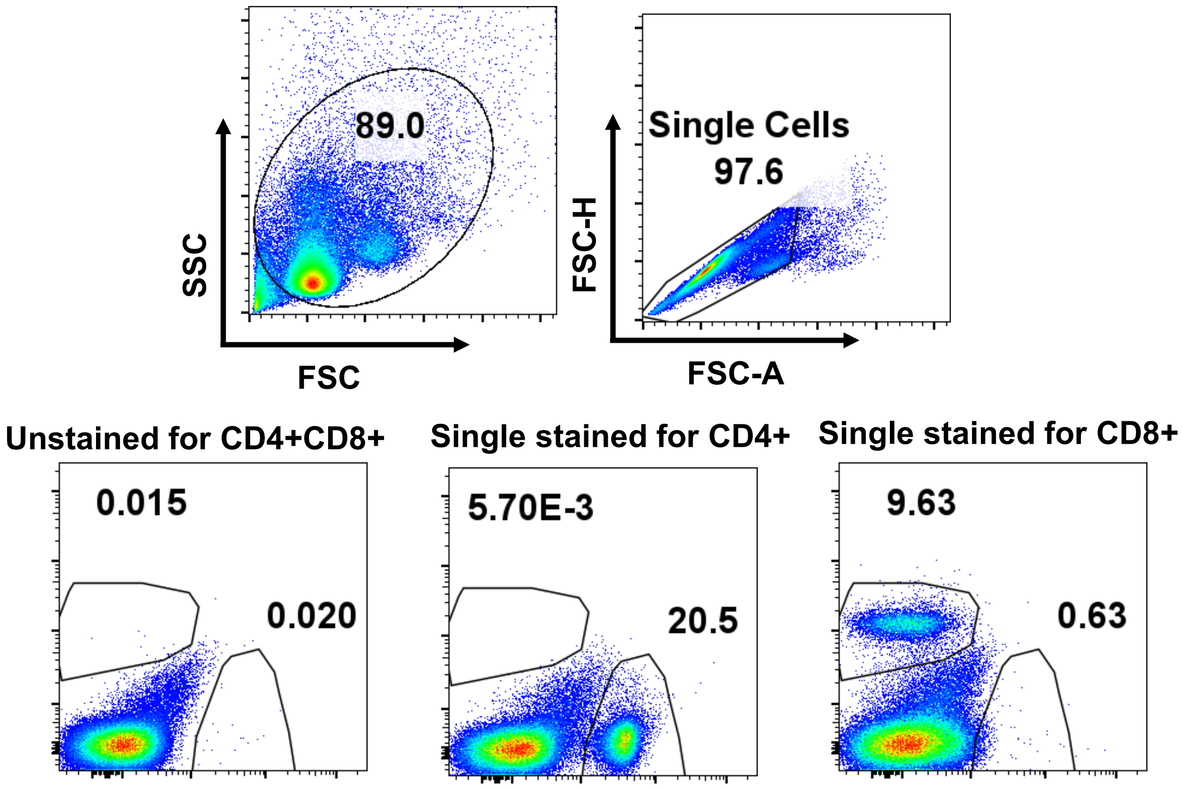

**Supplementary Table 1. Descriptive Statistics of the HC Population and *Spn* Population**

| | **All *HC*** |
|---|---|
| **Age** Median age (range) | 40 (29-61) |
| **Gender** (N, % male) | 5/10 (50%) |
| **Clinical diagnosis** | |
| Pneumonia | 0/10 (0%) |
| Shock | 0/10 (0%) |
| **ICU admission (N, %)** | 0/10 (0%) |
| | **All *Spn*** |
| **Age** Median age (range) | 47 (32-68) |
| **Gender** (N, % male) | 4/7 (57%) |
| **Clinical diagnosis** | |
| Pneumonia | 7/7 (100%) |
| Shock | 2/7 (29%) |
| **ICU admission (N, %)** | 4/7 (57%) |

| Mouse | CFU |
|---|---|
| #1 | 0 |
| #2 | 0 |
| #3 | 0 |
| #4 | 0 |
| #5 | 0 |
| #6 | 0 |
| #7 | 0 |

**Supplementary Table 2.** After 1 week of antibiotics, mouse hearts were homogenized in 1 mL PBS, serially diluted, and plated on Sheep's Blood Agar before incubating overnight at 37°C. Limit of detection is 100 CFU/mL of homogenate.

**Supplementary Figure 1. Post-Spn Infection Survived mice Show systemic Chronic Inflammation through Elevated innate immune cell (Myeloid Cell) Frequencies. (a)** Serum troponin-I measured by ELISA in control and Spn infected mice (N=4-10). **(b)** Representative TUNEL's staining in LV regions (50µm scale) and Quantification % TUNEL positive nuclei (N=4). **(c)** Representative H&E staining in LV regions (50µm scale) and quantification of cardiomyocytes area (N=4). (d) Representative figure of flow cytometry showing gating strategy to measure $CD11b^+$ myeloid cells from the spleen of all groups. **(e)** Data represents the quantitation of the percent of total $CD11b^+$ myeloid cells (N=11-18). **(f)** Representative figure of flow cytometry showing gating strategy to measure $CD11b^+F4/80^+$ macrophages from the spleen of all groups. **(g)** Data represents the quantitation of the percent of total macrophages (N=11-18). **(h)** Representative figure of flow cytometry showing the gating strategy to measure M1 macrophages from the spleen of all three groups. **(i)** Data represents the quantitation of the percent of total M1 macrophages (N=11-18). **(j)** Data represents the quantitation of the percent of total M2 macrophages (N=11-18). **(k)** Data represents the ratio of M1/M2 macrophages (N=12-18). **(l)** Representative figure of flow cytometry showing gating strategy to measure DCs from the spleen of all three groups. **(m)** Data represents the quantitation of percent of total DCs (N=12-18). **(n)** Representative figure of flow cytometry showing the gating strategy to measure neutrophils from the spleen of all three groups. **(o)** Data represents the quantitation of percent of total neutrophils (N=5-6)**.** *p<0.05, **p<0.01, ***p<0.001, ****p<0.0001, Data are presented as mean ± standard deviation and the significance of the data was determined using a two-tailed Mann-Whitney U test.

**Supplementary Figure 2.** Flow cytometry gating strategy for identifying pNF-$\kappa B^+$ myeloid cells and neutrophils, using unstained and single-stained controls.

**Supplementary Figure 3.** Flow cytometry gating strategy for identifying TNF-$\alpha^{+}$, IL-1β, and IL-6+ cells using unstained and single-stained controls.

**Supplementary Figure 4.** Flow cytometry gating strategy for identifying Th1 and Th2 cells using unstained and single-stained controls.

**Supplementary Figure 5.** Flow cytometry gating strategy for identifying Treg cells using unstained and single-stained controls.

**Supplementary Figure 6. Delayed Cell Death in Antigen-Specific Myeloid and T Cells.** Myeloid and T cells were sorted from control, antibiotic-treated, and infected-then-antibiotic-treated animals. These cells were co-cultured at a 10:1 ratio (T cells to myeloid cells) and stimulated with CSA (5 μg/mL) for 72 hours. Cytotoxicity was assessed using flow cytometry with Annexin V and 7AAD staining. **(a)** Representative flow diagram to show cell death in myeloid cells **(b)** % Apoptotic myeloid cells ($CD11b^{+}AnexinV^{+}7AAD^{+}$) (N=3) and **(c)** % Apoptotic T cells ($TCR\alpha\beta^{+}AnexinV^{+}7AAD^{+}$) (N=3). ****$p<0.0001$, Data are presented as mean ± standard deviation and the significance of the data was determined using a two-tailed Mann-Whitney U test.

**Supplementary Figure 7. Spn-survived animals exhibit activated antigen-specific T cells but show impairment in antigen-specific Tregs.** Myeloid and T cells were sorted from control, and infected-then-antibiotic-treated animals. These cells were co-cultured at a 10:1 ratio (T cells to myeloid cells) and stimulated with CSA (5 μg/mL) for 72 hours. BrdU was incorporated 24 hours before harvesting the samples for the experiment. **(a)** Representative flow diagram to show proliferation in myeloid cells **(b)** % of proliferated myeloid cells ($CD11b^{+}Brdu^{+}$) (N=7-16)and **(c)** Representative flow diagram to show proliferation in CD4+ T cells cells **(d)** % of proliferated

CD4+ T cells (TCRαβ+CD4+Brdu+) (N=8-18) **(e)** Representative flow diagram to show proliferation in CD8+ T cells cells **(f)** % of proliferated CD8+ T cells (TCRαβ+CD8+Brdu+) (N=8-18) **(g)** Representative flow diagram to show activation in CD4+ T cells cells **(h)** % of activated CD4+ T cells (TCRαβ+CD4+CD69+) (N=8-18) **(i)** Representative flow diagram to show activation in CD8+ T cells cells **(j)** % of activated CD8+ T cells (TCRαβ+CD8+CD69+)(N=8-18) **(k)** % of proliferated Treg cells (CD4+CD25+Foxp3+Brdu+)(N=8-17). ****p<0.0001, Data are presented as mean ± standard deviation and the significance of the data was determined using a two-tailed Mann-Whitney U test.

**Supplementary Figure 8. Increased B Cell Frequency but Reduced Anti-Inflammatory Cytokine Production in Spn-Survived Animals. (a)** Representative flow diagram to show CD19+ B cells in spleen **(b)** % of splenic B cells (CD19$^{+}$) (N=13-21)and **(c)** % of cardiac B cells (CD45+CD19+) (N=13-21) **(d)** Representative flow diagram to show IL10+ cells in spleen **(e)** % of IL10+ splenic (IL10+) cells (N=13-21) **(f)** % of TGFβ+ splenic (TGFβ+) cells (N=13-21) **(g)** % of CD19+IL10+ splenic (CD19+IL10+) cells (N=13-21) **(h)** % of CD19+TGFβ+ splenic cells (N=13-21). ****p<0.0001, Data are presented as mean ± standard deviation and the significance of the data was determined using a two-tailed Mann-Whitney U test.

**Supplementary Figure 9. Hydrocortisone decreases leukocyte infiltration in the heart following Spn infection.** IHC showing CD45$^{+}$ leukocytes (200µm scale).

**Supplementary Figure 10. Hydrocortisone Suppresses NF-ƙB Activation in Neutrophils. (a)** Representative figure of flow cytometry showing the gating strategy to measure pNF-ƙβ$^{+}$ neutrophils from the spleen of all three groups. **(b)** Data represents the quantitation of the percent of total neutrophil cells (N=6-7). **(c)** Data represents the quantitation of the percent of total pNF-ƙβ$^{+}$ neutrophils (N=6-7). **(d)** Flow cytometry gating strategy for identifying pNF-κB$^{+}$ neutrophils,

using unstained and single-stained controls. *p<0.05, **p<0.01, ***p<0.001, ****p<0.0001, Data are presented as mean ± standard deviation and the significance of the data was determined using One Way ANOVA.

**Supplementary Figure 11. Hydrocortisone suppresses NF-ƙB activation in myeloid cells. (a)** Representative figure of flow cytometry showing the gating strategy to measure pNF-ƙβ$^{+}$ myeloid cells from the spleen of all three groups. **(b)** Data represents the quantitation of the percent of total myeloid cells (N=6-7). **(c)** Data represents quantitation of percent of total pNF-ƙβ$^{+}$ myeloid cells (N=6-7). **(d)** Flow cytometry gating strategy for identifying pNF-κB$^{+}$ myeloid cells using unstained and single-stained controls. *p<0.05, **p<0.01, ***p<0.001, Data are presented as mean ± standard deviation and the significance of the data was determined using One Way ANOVA.

**Supplementary Figure 12. Hydrocortisone Suppresses NF-ƙB Activation in Macrophages. (a)** Representative figure of flow cytometry showing the gating strategy to measure pNF-ƙβ$^{+}$ macrophages from the spleen of all three groups. **(b)** Data represents the quantitation of the percent of total macrophages (N=6-7). **(c)** Data represents the quantitation of the percent of total pNF-ƙβ$^{+}$ macrophages (N=6-7). **(d)** Flow cytometry gating strategy for identifying pNF-κB$^{+}$ macrophages using unstained and single-stained controls. **p<0.01, ***p<0.001, Data are presented as mean ± standard deviation and the significance of the data was determined using One Way ANOVA.

**Supplementary Figure 13. Hydrocortisone inhibits pro-inflammatory cytokines. (a)** Representative figure of flow cytometry showing the gating strategy to measure IL-1β$^{+}$ cells from the spleen of all three groups. **(b)** Data represents the quantitation of the percent of total IL-1β+ cells (N=6-7). **(c)** Representative figure of flow cytometry showing the gating strategy to measure IL-6$^{+}$ cells from the spleen of all three groups. **(d)** Data represents the quantitation of the percent

of total IL-6+ cells (N=6-7). **(e)** Representative figure of flow cytometry showing the gating strategy to measure TNF-$\alpha^+$ cells from the spleen of all three groups. **(f)** Data represents the quantitation of the percent of total TNF-$\alpha^+$ cells (N=6-7). *p<0.05, **p<0.01, ***p<0.001, Data are presented as mean ± standard deviation and the significance of the data was determined using One Way ANOVA.

**Supplementary Figure 14.** Flow cytometry gating strategy for identifying TNF-$\alpha^+$, IL-1β, and IL-6+ cells using unstained and single-stained controls.

**Supplementary Figure 15. Paquinimod treatment does not affect morphometric changes.** Shown are **(a)** Body weight (N=9-12), **(b)** morphometric changes (N=11-12) **(c)** cardiomyocyte area (N=3-4).

**Supplementary Figure 16. Paquinimod treatment does not affect M2 polarization but promotes Treg differentiation. (a)** Representative figure of flow cytometry showing gating strategy to demonstrate M2 macrophages from the spleen of all three groups. **(b)** Data represents the quantitation of the percent of total M2 macrophages cells (N=5-8). **(c)** Representative figure of flow cytometry showing gating strategy to demonstrate CD4$^+$ and CD8+ cells from the spleen of all three groups. **(d)** Data represents the quantitation of the percent of total CD4+ T cells (N=5-7). **(e)** Data represents the quantitation of the percent of total CD4+CD25+ T cells (N=6-7). **(f)** Data represents the quantitation of percent of total CD4+CD69+ T cells (N=6-8). **(g)** Data represents the quantitation of the percent of total Treg cells (N=6-7). **(h)** Data represents quantitation of percent of total CD8+ T cells (N=5-8) **(i)** Data represents quantitation of percent of total CD8+CD69+ T cells (N=6-7). *p<0.05, **p<0.01, ***p<0.001, Data are presented as mean ± standard deviation and the significance of the data was determined using One Way ANOVA.

**Supplementary Figure 17. (a)** Flow cytometry gating strategy for identifying M2 macrophages cells using unstained and single-stained controls. **(b)** Flow cytometry gating strategy for identifying CD4 and CD8+ T cells using unstained and single-stained controls.
